# Multiple system atrophy-associated oligodendroglial protein p25α stimulates formation of novel α-synuclein strain with enhanced neurodegenerative potential

**DOI:** 10.1007/s00401-021-02316-0

**Published:** 2021-05-12

**Authors:** Nelson Ferreira, Hjalte Gram, Zachary A. Sorrentino, Emil Gregersen, Sissel Ida Schmidt, Lasse Reimer, Cristine Betzer, Clara Perez-Gozalbo, Marjo Beltoja, Madhu Nagaraj, Jie Wang, Jan S. Nowak, Mingdong Dong, Katarina Willén, Ersoy Cholak, Kaare Bjerregaard-Andersen, Nicolas Mendez, Prakruti Rabadia, Mohammad Shahnawaz, Claudio Soto, Daniel E. Otzen, Ümit Akbey, Morten Meyer, Benoit I. Giasson, Marina Romero-Ramos, Poul Henning Jensen

**Affiliations:** 1 DANDRITE, Danish Research Institute of Translational Neuroscience & Department of Biomedicine, Aarhus University, 8000 Aarhus C, Denmark; 2 Department of Neuroscience, Center for Translational Research in Neurodegenerative Diseases and McKnight Brain Institute, University of Florida, Gainesville, USA; 3 Department of Neurobiology Research, Institute of Molecular Medicine, University of Southern Denmark, J.B. Winsloews Vej 21, st, 5000 Odense C, Denmark; 4 Interdisciplinary Nanoscience Center (iNANO), Aarhus University, 8000 Aarhus C, Denmark; 5 Institute for Advanced Materials, School of Material Science and Engineering, Jiangsu University, Zhenjiang, 212013 China; 6 Department of Cell Biology, H. Lundbeck A/S, Valby, Denmark; 7 Mitchell Center for Alzheimer’s Disease and Related Brain Disorders, Department of Neurology, University of Texas McGovern Medical School At Houston, Houston, TX USA; 8 Aarhus Institute of Advanced Studies (AIAS), Aarhus University, 8000 Aarhus C, Denmark; 9 Institute of Complex Systems (ICS6), Structural Biochemistry, Research Center Julich, 52415 Julich, Germany; 10 BRIDGE, Brain Research, Inter-Disciplinary Guided Excellence, Department of Clinical Research, University of Southern Denmark, J.B. Winsloews Vej 19, 5000 Odense C, Denmark

**Keywords:** Α-Synuclein, P25α, Tubulin polymerisation-promoting protein (TPPP), Strains, Multiple system atrophy (MSA), Protein aggregation

## Abstract

Pathology consisting of intracellular aggregates of alpha-Synuclein (α-Syn) spread through the nervous system in a variety of neurodegenerative disorders including Parkinson’s disease, dementia with Lewy bodies, and multiple system atrophy. The discovery of structurally distinct α-Syn polymorphs, so-called strains, supports a hypothesis where strain-specific structures are templated into aggregates formed by native α-Syn. These distinct strains are hypothesised to dictate the spreading of pathology in the tissue and the cellular impact of the aggregates, thereby contributing to the variety of clinical phenotypes. Here, we present evidence of a novel α-Syn strain induced by the multiple system atrophy-associated oligodendroglial protein p25α. Using an array of biophysical, biochemical, cellular, and in vivo analyses, we demonstrate that compared to α-Syn alone, a substoichiometric concentration of p25α redirects α-Syn aggregation into a unique α-Syn/p25α strain with a different structure and enhanced in vivo prodegenerative properties. The α-Syn/p25α strain induced larger inclusions in human dopaminergic neurons. In vivo*,* intramuscular injection of preformed fibrils (PFF) of the α-Syn/p25α strain compared to α-Syn PFF resulted in a shortened life span and a distinct anatomical distribution of inclusion pathology in the brain of a human A53T transgenic (line M83) mouse. Investigation of α-Syn aggregates in brain stem extracts of end-stage mice demonstrated that the more aggressive phenotype of the α-Syn/p25α strain was associated with an increased load of α-Syn aggregates based on a Förster resonance energy transfer immunoassay and a reduced α-Syn aggregate seeding activity based on a protein misfolding cyclic amplification assay. When injected unilaterally into the striata of wild-type mice, the α-Syn/p25α strain resulted in a more-pronounced motoric phenotype than α-Syn PFF and exhibited a “tropism” for nigro-striatal neurons compared to α-Syn PFF. Overall, our data support a hypothesis whereby oligodendroglial p25α is responsible for generating a highly prodegenerative α-Syn strain in multiple system atrophy.

**Supplementary Information:**

The online version contains supplementary material available at 10.1007/s00401-021-02316-0.

## Introduction

Multiple system atrophy (MSA), along with Parkinson’s disease (PD) and dementia with Lewy bodies (DLB) are progressive neurodegenerative disorders. They share the development of intracellular inclusions containing aggregates of the nerve cell protein alpha-Synuclein (α-Syn) and are often collectively referred to as synucleinopathies [31]. α-Syn is a small presynaptic protein involved in SNARE complex functionality and synaptic vesicle release [12, 101]. In synucleinopathies, α-Syn turns into insoluble aggregated species hyperphosphorylated on serine129 that deposits in cytoplasmic inclusions [2, 26]. α-Syn is considered a key player in the synucleinopathies, because missense mutations and gene multiplications in the SNCA gene cause autosomal dominant familial PD and DLB, and GWAS studies demonstrate variations in the α-Syn-encoding SNCA locus, which is a risk factor for PD [40]. The pathology of synucleinopathies is hypothesised to be spreading through the nervous tissue by native α-Syn turning into aggregated prion-like species that upon spreading from one cell to a recipient cell templates the native α-Syn into toxic aggregates that can perpetuate the process [11, 47, 56]. Experimental evidence supports this hypothesis by demonstrating that inoculation of α-Syn aggregate-containing extracts from animals, patient brains, and in vitro preformed α-Syn fibrils (PFF) into the nervous system of laboratory animals initiates aggregation of endogenous α-Syn in nerve cells and subsequent spreading of α-Syn pathology into anatomically connected areas [23, 61, 62, 105].

MSA has a faster disease progression than other synucleinopathies and differs from them pathologically [97]. In PD and DLB, α-Syn aggregates accumulate in Lewy bodies (LB) that reside in α-Syn-expressing neurons [31, 66]. This contrast to MSA, where the burden of α-Syn aggregates resides in glial cytoplasmic inclusions (GCI) in oligodendrocytes [27, 96], but also accumulates in neurites and neuronal cell bodies [27, 36, 66, 90, 96]. How the large amounts of α-Syn accumulate in the oligodendrocytes is still unresolved [17]. An early in situ hybridization study could not demonstrate any expression [70], but a recent qPCR analysis of oligodendrocytes isolated from human brain demonstrated α-Syn mRNA in oligodendrocytes [4]. Moreover, oligodendroglial precursor cells and in vitro cultured human and rodent oligodendroglial cells express α-Syn mRNA and protein [18, 44, 65]. Treatment of oligodendrocytes and its precursors with exogenous α-Syn aggregates increases the low level of endogenous α-Syn by templating the formation of stable α-Syn aggregates [44, 44, 65], and this has been hypothesised to represent a neuron–oligodendroglial pathway of importance for MSA [43]. MSA brain extracts and in particular the detergent-insoluble fraction containing the α-Syn filaments are more potent seeds of α-Syn aggregation in cellular and in vivo models [79, 89, 108, 114, 115]. This potency can be propagated in laboratory animals and is one reason why MSA has been proposed to represent a distinct prion-like disease [79, 108, 114, 115]. Mounting evidence supports that the α-Syn aggregation in oligodendrocytes represents the key to MSA brain extracts being potent inducers of α-Syn aggregation in disease models [77].

α-Syn filaments isolated from MSA brains possess unique structures as demonstrated by cryoelectron microscopy [89], and α-Syn aggregates in human GCI and LB can be distinguished by specific antibodies [65, 77]. The differences observed between the distinct α-Syn inclusion types are recapitulated in the aggregates generated in α-Syn mouse models overexpressing α-Syn in oligodendrocytes compared to mice only expressing α-Syn in neurons upon initiation of aggregation by intracranial injection of α-Syn PFF [77]. Surprisingly, α-Syn-expressing oligodendrocytes were able to phenoconvert LB-like α-Syn aggregates to make GCI-like inclusions, but neurons could not phenoconvert GCI-like strains to make LB-like inclusions [77]. This suggests that factors in the oligodendroglial milieu determine how MSA-associated α-Syn strains are generated [77]. We hypothesise that the oligodendroglial protein p25α, also named tubulin polymerization promoting protein (TPPP), could be one such factor. P25α in oligodendrocytes converts their low amounts of cellular α-Syn into stable, insoluble, and toxic species [49, 50, 65], and p25α stimulates aggregation of α-Syn in vitro [58]. In MSA, p25α becomes dyslocalized from myelin and the nucleus into the perinuclear oligodendroglial cytosol prior to the accumulation of α-Syn in GCI where the two proteins colocalize [58, 74, 83, 94]. In synucleinopathies, p25α also accumulates in neurons, whereas in MSA, it occurs in both α-Syn-negative and α-Syn-positive cytoplasmic and nuclear inclusions [6]. In PD, the two proteins colocalise in some LB [48, 58, 83], thereby increasing the likelihood of aberrant interactions between the two proteins. We hypothesise that the encounter between p25α and dystopic α-Syn in oligodendrocytes and dystopic p25α with α-Syn in neurons causes the formation of a special strain of p25α-induced α-Syn aggregates, aggregates that are responsible for the rapid and fatal neurodegeneration in MSA.

In the present work, we generated a novel α-Syn strain by incubating α-Syn in the presence of p25α, and compared this α-Syn/p25α PFF to a control α-Syn fibril strain made in the absence of p25α. Structurally, the two strains differed on biochemical, biophysical, and structural parameters. Functionally, the p25α strain was more effective in templating α-Syn aggregation in human neuronal stem cell-derived dopaminergic neurons. When inoculated into wild-type and heterozygous A53T-α-Syn M83 transgenic mouse models, α-Syn/p25α PFF templates inclusions with a different morphology during their spreading in the CNS and cause a more rapid disease course.

Analysis of brain stem extracts of end-stage heterozygous A53T-α-Syn M83 mice demonstrated an increased amount of aggregates in α-Syn/p25α PFF-injected mice as determined by a Förster resonance energy transfer immunoassay, and is in agreement with the more rapid disease course compared to control α-Syn PFF. Surprisingly, we observed a lower level of templating active seeds in the extracts from the α-Syn/p25α PFF-injected mice using a protein misfolding cyclic amplification assay. Our findings suggest that the α-Syn/p25α aggregates template α-Syn with an enhanced neurodegenerative potential and demonstrate that p25α is a strong candidate as an oligodendroglial factor responsible for generation of MSA α-Syn strains.

## Methods

### Production and purification of α-Syn and p25α

Full-length human wild-type and S129A-mutant α-Syn (1–140) proteins were expressed in BL21(DE3)-competent cells and purified as previously described [22, 58]. Briefly, α-Syn purification involved dialysis of heat-stable *E. coli* extracts against 20 mM Tris pH 6.5 overnight, followed by ion-exchange chromatography on a Poros HQ50 column (Thermo Fisher Scientific) with a 0–2 M NaCl gradient in the dialysis buffer. This was followed by an additional reverse phase-high pressure liquid chromatography purification step on a Jupiter C18 column (Phenomenex, Torrance, CA) in 0.1% trifluoroacetic acid with a 0–90% acetonitrile gradient. Isolated α-Syn was then extensively dialysed against PBS pH 7.4 overnight followed by an additional dialysis step against 20 mM ammonium bicarbonate overnight. The latter dialysis was performed, because it allows the subsequent lyophilization to remove the volatile ammonium bicarbonate and leave pure protein without any salt for storage. The protein concentration was determined by bicinchoninic acid (BCA) protein concentration assay (Pierce). The proteins were subsequently aliquoted, lyophilized, and stored at − 80 °C until use. The human p25α was expressed in BL21(DE3)-competent cells and purified as previously described [58]. Briefly, the heat-soluble proteins were purified on a Poros HS50 cation exchange column (PerSeptive Biosystems, Foster City, CA) followed by gel filtration on a GF-75 gel filtration column (Amersham Biosciences).

### Quantitative α-Syn fibril assembly and sedimentation

Soluble monomeric wild-type and S129A α-Syn (346 μM) were assembled in the absence or presence of p25α (17 μM) into preformed fibrils (PFF) by incubation at 37 °C in phosphate-buffered saline pH 7.4 (PBS, Gibco) with continuous shaking at 1050 r.p.m. (Eppendorf Thermotop) for 72 h. The generated PFF were harvested by centrifugation 15,600 *g* at 25 °C for 30 min and then resuspended in PBS to a concentration of 2 mg/mL, as determined by BCA protein concentration assay (Pierce) using 0.1 M NaOH as diluent to completely dissociate the PFF. Then, PFF were sonicated for 20 min using a Branson 250 sonifier at 30% intensity before being aliquoted and frozen at − 80 °C until use. A fraction of each sample was set aside for K114 fluorometry [14]. The remainder of each sample was centrifuged at 100,000 *g* for 20 min. SDS sample buffer (10 mM Tris, pH 6.8, 1 mM EDTA, 40 mM DTT, 1% SDS, 10% glycerol) was added to pellets and supernatants, which were heated to 96 °C for 15 min. Equal volumes of α-Syn proteins in the supernatants and pellets were separated by SDS-PAGE; gels were scanned and quantified using Image J software (National Institutes of Health, Bethesda, MD, USA).

### K114 and thioflavin T fluorometry

To quantify the amount of amyloid formation, samples were monitored by (trans,trans)-1-bromo-2,5-bis-(4-hydroxy)styrylbenzene (K114) or thioflavin T (ThT) fluorometry as described previously [14]. In brief, samples were analysed by incubating a fraction of each sample with K114 (50 µM) in 100 mM glycine, pH 8.5, and measuring fluorescence (*λ*_ex_ = 380 nm, *λ*_em_ = 550 nm). For ThT analysis, samples were incubated with ThT (20 µM) in 90 mM glycine, 0.01% Triton X-100, pH 8.5, and fluorescence measured (*λ*_ex_ = 450 nm, *λ*_em_ = 482 nm). Both ThT and K114 fluorescence were measured with an EnSpire 2300 Multilabel Reader (Perkin Elmer).

### Atomic force microscopy (AFM)

In AFM measurement, muscovite mica was freshly cleaved by tape and used as substrate. PFF, 8 times diluted at a volume of 10 μL, were deposited on freshly cleaved mica for 30 min. Then, the excess sample was removed, and the mica was rinsed once with Milli-Q water and later dried in ambient condition for 1 h before measurement. The AFM morphology measurements were completed using an equipment (Multimode 8, Bruker Co., Ltd., USA) in tapping mode with ultra-sharp silicon cantilevers (OMCL-AC160TS-R3; Olympus). During the measurements, the scan rate was set at 1 Hz. Resonant frequency was set at 300 kHz. The resolution of all AFM images was 512 × 512 pixels.

### Transmission electron microscopy (TEM)

The grids used for measurement were made of copper with 200–400 mesh spacing. Three microliters of PFF (2 mg/ml) were placed on the grid for 1 min, and sample excess was then removed using filter paper. Then, each grid was stained with 3 μL of staining solution (2% uranyl acetate in water) for 30 s. After the excess sample was removed, grids were washed with Milli-Q water (3 × 10 μL) and allowed to dry at room temperature. Finally, the grids were visualised with a Tecnai EM microscope equipment (Tecnai G2, FEI, USA).

### Circular dichroism (CD)

Far-UV CD spectra were recorded on a Chirascan-plus CD spectrophotometer (Applied Photophysics, U.K.) using a 1 mm quartz cuvette at a concentration of 0.4 mg/ml protein. PFF were sonicated beforehand with a Q500 sonicator (Qsonica, Connecticut) for 10 s at 20% intensity, using a 1.6 mm Microtip Probe. The samples were measured using a step size of 0.5 nm, 0.5 s per point, and 1 nm bandwidth. For each sample, three spectra were recorded and averaged.

### Fourier-transform infrared spectroscopy (FTIR)

FTIR spectra were recorded on a Tensor 27 FTIR (Bruker, Massachusetts USA). The samples from CD measurements were recycled and spun at 13.5 Kr.p.m. for 5 min. Three microliter samples from the bottom of the Eppendorf tubes were resuspended, transferred onto the quartz crystal, and dried gently with nitrogen. Sixty-eight scans were averaged (1000–3998 cm^−1^, resolution of 2 cm^−1^). OPUS software was used for baseline correction, atmospheric compensation, second-derivate calculation, and spectral normalisation.

### Dynamic light scattering (DLS)

After α-Syn sedimentation, PFF were diluted to 2 mg/ml in sterile PBS pH 7.4 (Gibco) and subjected to ultrasound breakage for 20 min using a Sonifier (Branson 250; 30% intensity) equipped with a water jacket cooling system to avoid sample overheating. Then, the size distribution profile of PFF in suspension was measured by DLS using a Wyatt DynaPro NanoStar instrument at 25 °C. Data were processed using the Dynamics 7.5.0.17 software package with the solvent (PBS) background signal subtracted from each sample.

### Nuclear magnetic resonance spectroscopy (NMR)

Solid protein samples were packed into 3.2 mm Bruker rotors, ~ 10 mg. The high-resolution NMR spectra were recorded using a Bruker 950 MHz NMR spectrometer. MAS frequency was set to 13.5 kHz and the samples were temperature regulated at 270 K. Spectra were processed using the Topspin 3.5 processing program. 2D ^13^C-^13^C PDSD spectrum represented in Fig. 2 was recorded with a 20 ms mixing period and ~ 5 ms indirect dimension evolution time. Three seconds of recycle delay was used and 128 transients were acquired. The spectra were processed with a shifted squared sine-bell window function of 3.

### Proteolytic digestion of α-Syn strains

Parental PFF (G0) were obtained as mentioned above. Second (G1) and third (G2) generations of α-Syn or α-Syn/p25α PFF were made by seeding wild-type α-Syn monomers (69 µM, in PBS pH 7.4) with 3.5 µM of G0 or G1 fibrils, respectively. Samples were then incubated for 5 days at 37 °C under agitation (1050 r.p.m., Eppendorf Thermotop) and then purified as previously described.

*PFF digestion*: Digestion of α-Syn and α-Syn/p25α PFF was performed by incubating PFF (0.8 mg/mL) with 0, 2.5, 5, or 20 µg/mL proteinase K (Sigma-Aldrich) in digestion buffer (78 mM NaCl, 12 mM Tris, 6 mM CaCl_2_, pH 7.8). After 30 min of incubation at 37 °C and shaking at 400 r.p.m., the reaction was stopped by addition of 0.02 mM Pefabloc (Sigma-Aldrich) for 10 min. Samples were then processed for gel electrophoresis.

*Gel electrophoresis and Western blot:* Loading buffer (100 mM Tris–HCl, 8% SDS, 24% glycerol, 0.02% bromophenol blue, and pH 6.8) was added to the samples 1:1; then, samples were denatured at 95 °C for 2 × 5 min. After centrifugation for 5 min at 16,300 r.p.m., the supernatant corresponding to 12 µg protein was loaded into 16% Tricine gels (Novex) or 8–16% polyacrylamide gels (GenScript). Protein bands were stained with Coomassie Blue (0.25% Coomassie Brilliant Blue G-250, 40% EtOH, 10% acetic acid) or blotted into PVDF membranes using iBlot® 2 Dry Blotting System (Thermo Fischer). The membranes were then fixed with 4% PFA in PBS for 30 min and then boiled in PBS for 5 min. After being blocked for 30 min (TBS, 0.05% Tween, skimmed milk powder, pH 7.6), membranes were incubated with primary antibodies, mouse monoclonal anti-α-Syn Syn-1 (BD Biosciences #610,787, 1:1000), or mouse monoclonal anti-α-Syn LB509 (abcam #27,766, 1:1000) ON at 4 °C. Membranes were subsequently incubated with secondary horseradish peroxidase-conjugated anti-mouse (Dako, Denmark) for 1.5 h at RT. Protein bands were visualised with ECL (GE Healthcare, UK) and image acquisition by Fuji Las-3,000 intelligent dark box (Fujifilm, Japan).

### Dot-blot

Samples were spotted by non-denaturing immuno-dot blot as previously described [53]. Briefly, different amounts of PFF (3.12–100 ng), monomeric α-Syn (100 ng), or vehicle (PBS) were dotted directly onto a nitrocellulose membrane using a vacuum filtration system (Bio-Rad Bio-Dot Apparatus). Membranes were incubated with primary antibodies anti-α-Syn Syn-1 (BD Biosciences #610,787, 1:1000), anti-p25α (produced in house [58], affinity rabbit polyclonal, 1:1000), and aggregate-specific MJF14-6–4-2 (MJF14) (Abcam #209,538, 1:45,000) and fibril-specific FILA-1 (produced in house [59], 1:1,000). Proteins were visualised using ECL in a Fuji LAS-3000 Intelligent Dark Box (Fujifilm, Japan).

### Cell cultures

*Human iPSC-derived neural stem dopaminergic neurons*: Induced pluripotent stem cell (iPSCs)-derived dopaminergic neurons were differentiated for 45 days using a DOPA differentiation kit (XCell Science) [9]. At day 38 of differentiation, neurons were incubated with S129A-α-Syn or S129A-α-Syn/p25α PFF (14 µg/ml) in cell medium for 24 h. S129A-α-Syn PFF was used in the cellular experiments to avoid any pS129-α-Syn signal to arise from phosphorylation of the exogenous aggregates. Untreated neurons were included as control. After 24 h, cells were carefully washed in PBS and allowed to grow for an additional 6 days in fresh medium before fixation in 4% PFA. Two hours prior to fixation, cells were treated with the polo like kinase inhibitor BI2536 (1 µM) to remove physiological nuclear pSer129 α-Syn immunoreactivity. The coverslips were subjected to immunostaining using primary antibodies against MAP2 (1:2000, Abcam ab92439) and pS129 (mouse monoclonal 11A5 kindly provided by Imago Pharmaceuticals 1:10,000) with the appropriate secondary Alexa Flour antibodies and DAPI. Five random pictures per coverslip were taken using a motorised stage on a Zeiss Observer.Z1 microscope with an X63 objective. Inclusion analyses were performed using ImageJ with inclusion size distributions; small (25–100 pixel^2^), medium (100–300 pixel^2^), and large (> 300 pixel^2^). All inclusions were measured as round objects > 25 pixel. Average values were taken for all five pictures per coverslip, and six coverslips per group from two independent differentiations (*n* = 6) were presented as mean ± SEM.

*Rat oligodendroglial cell line*: OLN-AS7 [80] stably expressing human α-Syn was maintained at 37 °C and 5% CO2 in DMEM supplemented with 10% foetal calf serum (FCS), 50 U/mL, 50 μg/mL penicillin/streptomycin (pen/strep), and 0.1 mg/mL Zeocin to select for α-Syn expression. α-Syn and α-Syn/p25 PFF (14 μg/ml) were added to the cell medium for 12 h to allow PFF uptake and intracellular templating. Then, cells were carefully washed trice with Hank's balanced salt solution (HBSS) to remove excess PFF, and fresh medium was added. Cells were allowed to grow for 36 h before being washed and fixed in 4% PFA and processed for ICC staining. The coverslips were subjected to immunostaining using primary antibodies against tubulin (1:5000, Abcam #ab6160) and pSer129 α-Syn (1:1000, Cell Signaling Technology #23,706) with the appropriate secondary Alexa Flour antibodies and 4′,6-diamidino-2-phenylindole (DAPI) (1:1000, Th.Geyer, 5 mg/mL). Ten pictures/coverslip were taken randomly using a motorised stage on a Zeiss Observer.Z1 microscope. Inclusion analyses were performed using ImageJ.

**In vivo induction and transmission of α-Syn pathology by intramuscular injection of α-Syn PFF.**

*Animals, mice treatments, and sample preparations*: Animals were housed in a temperature-controlled room under a 12 h light/dark period with water and food ad libitum. Three-month-old TgM83^+/−^ α-Syn mice were bilaterally injected with 5 µl solution (2 mg/ml) containing 10 µg of PFF, or vehicle (PBS, pH 7.4), by inserting the needle ∼1 mm deep into the gastrocnemius muscle as described elsewhere [86]. Mice were anaesthetised with isoflurane (3.5%) inhalation. Injections were made using different 10-μL Hamilton syringes with a 25-gauge needle to avoid any cross-contamination. Hindlimb clasping behaviour was monitored and scored regularly as previously reported [35, 41]. Once mice displayed hindlimb paralysis, they were sacrificed with pentobarbital euthanization and perfused with PBS with phosphatase inhibitors (25 mM β-glycerolphosphate, 5 mM NaF, 1 mM Na_3_VO_4_, and 10 mM Na-pyrophosphate) before removing the brain. Mice injected with vehicle (PBS, pH 7.4) did not develop any signs of the disease throughout the course of the experiment, and they were sacrificed at 180 d.p.i. Brains were fixed in 4% PFA in PBS for 48 h, and then stored on 30% sucrose in PBS with 0.05% sodium azide solution for IHC tissue processing.

*Immunohistochemistry*: Immunostaining of the sections was performed using well-established methods [19]. Sections were rehydrated, and subsequent antigen retrieval was performed in a steam bath for 30 min in a 0.2% Tween-20 solution for all antibodies utilised with the exception of cd11b where a modified citrate buffer (Target Retrieval Solution Citrate pH 6; Agilent, Santa Clara, CA) was used. Non-specific antibody binding was minimised with a 2% FBS/0.1 M tris block solution; primary antibodies were diluted in block solution and applied to tissue sections at 4 °C overnight. Biotinylated secondary antibodies (Vector Laboratories; Burlingame, CA) were similarly diluted in block solution and applied to sections for 1 h at room temperature. An avidin–biotin complex (ABC) system (Vectastain ABC Elite kit; Vector Laboratories, Burlingame, CA) was used to enhance detection of the immunocomplexes, which were visualised using the chromogen 3,3′-diaminobenzidine (DAB kit; KPL, Gaithersburg, MD). For each antibody, all slides were stained and DAB was exposed on the same run with intermixing of slides from each cohort throughout, so that there was no preferential DAB staining of one cohort vs the other in the selective antibodies. Furthermore, in the DAB staining the time to exposure was determined using a control slide, and the experimental slides were not examined until after coverslipping and light microscopy observation. Tissue sections were counterstained with haematoxylin. All slides were digitally scanned using an Aperio ScanScope CS instrument (40 × magnification; Aperio Technologies Inc., Vista, CA), and images of representative areas of pathology were captured using the ImageScope software (40 × magnification; Aperio Technologies Inc.).

Quantitation of 81a and 2H6 positivity was performed on both the brain stem and red nucleus for all cohorts. For each, mouse sections were analysed using the positive pixel count algorithm (Aperio, Leica Biosystems) as described previously [95], with the same intensity threshold values for all sections. One-way ANOVA with multiple comparison was used to determine significant differences (*p* < 0.05).

*Antibodies*: A panel of antibodies against α-Syn phosphorylated at Ser129 including 81A, EP1536Y, and LS4-2G12 was used as previously described [84, 110]. A number of mouse monoclonal N-terminal (1F11, 1D12, 9C10, 2H6, syn506) [15, 20, 109] or C-terminal (94-3A10, 15-4E7, 94-3B2) [15] antibodies of α-Syn were also used as they display differing affinities for various types of α-Syn inclusions [15, 16]. Other antibodies utilised for immunohistochemistry included those against p62/sequestosome (ProteinTech), Ubiquitin-1 (EMD Millipore), anti-pThr205 tau antibody 7F2 [100], GFAP (Wako), and cd11b (AbCam).

### In vivo induction and transmission of α-Syn pathology by intrastriatal injection of α-Syn PFF

*Animals* Female C57BL/6 mice (Janvier, *n* = 59), 6 weeks of age at the time of surgery, were housed six per cage under a 12 h light/12 h dark cycle at an average temperature of 21 °C with ad libitum access to food and water. All procedures involving animals were conducted under humane conditions, were approved by the Danish Animal Experiments Inspectorate (AEI), and complied with Danish laws and regulations for the Humane Care and Use of Animals in Research.

*Stereotaxic surgery* The animals were deeply anaesthetised with a combination of medetomidine, midazolam, and fentanyl i.p., and then placed in a stereotaxic frame with a mouse/newborn inset (Stoelting, Wood Dale, IL, USA). Two µl solution containing 10 µg of α-Syn solutions (Monomers *n* = 19, α-Syn PFF *n* = 20, and α-Syn/p25α PFF *n* = 20) were injected into the right striatum (coordinates, AP 0.7 mm, ML − 2.5 mm from bregma, and DV − 3.0 mm from dura), at a rate of 0.2 µl/30 s, using a glass cannula kept at the target position for another 5 min and then slowly retracted. The animals were sutured; pain relief was ensured with subcutaneous injection of an anti-sedative mix of naloxone, atipamezole, and flumazenil, and a solution of buprenorphine. When completely awake, the mice were returned to the home cage.

### Behavioural tests  

*Challenging beam traversal test* This test is useful for evaluating motor performance and coordination deficits. It was performed at 3 and 6 months after surgery as previously described [25]. Briefly: the beam consists of four 25-cm-long frames of decreasing width (3.5, 2.5, 1.5, and 0.5 cm, respectively) and a removable grid on top of the beam. Two inverted clean cages are used to support the beam, and the home cage is placed at the narrow end of the beam. Mice were trained to transverse the beam from the widest to narrowest frame for 2 days without the grid on. The trials end when the mouse places one of its forelimbs into the home cage. On the 3rd day, the grid is placed on top of the beam and a video camera is used to record all the trials. All mice transverse the grid surface beam five times. The videos were analysed by an observer, blinded to the animal’s identity, recorded time to traverse each frame, time to enter the cage, and errors (paw slips through the grid) and steps in frames 3 and 4. The data used are the average of the five trials.

*Cylinder test* Mice were tested 3 and 6 months after surgery to evaluate locomotor asymmetry using the cylinder test as described previously [25]. Briefly, the animals are placed in a transparent Plexiglas cylinder (diameter 20 cm) with a mirror placed behind it to visualise the cylinder surface fully. A camera is placed in front and adjusted until the whole bottom of the cylinder can be seen in the video from the mirror. Spontaneous use of the animal’s forepaws (independently or simultaneously) was video recorded during 3 min or until the number of vertical activity/rearing was 20. The experiment was conducted in a dark room with a red light as the only light source. The videos were analysed by an observer, blinded to the mouse’s identity, who recorded right and left forelimb and hindlimb steps. Data are reported as the percentage of contralateral limb use: [(contra + ½ both) divided by (ipsi + contra + both)] × 100.

*Histology* Animals were euthanised by a pentobarbital overdose and upon respiratory arrest, and perfused with physiological saline (0.9% saline) through the ascending aorta followed by ice-cold 4% PFA (50–75 ml per mouse). The brain was carefully removed and post-fixed in the same PFA solution for 2 h and cryoprotected in a 25% sucrose solution until processing. The brains were sectioned into 40-μm-thick serial coronal sections using a freezing microtome (Microm HM 450, Brock&Michelsen, Denmark) in series of six for the forebrain (striatum) and four for the midbrain, and stored in an antifreeze solution at – 20 °C until further processing. Immunohistochemical staining was performed on free-floating brain sections using the following primary antibodies: TH (AB152, Rabbit polyclonal, Millipore 1:750), aggregated α-Syn (MJF14 (Abcam #209538, 1:25,000), Iba1 (019–19,741, Wako, Rabbit polyclonal 1:800), GAP (ab7260, Abcam, Rabbit polyclonal, 1:5000), MHC II (14–5321, Rat monoclonal, eBioscience 1:400), and CD68. After incubation with appropriate secondary antibodies, sections were washed and incubated with avidin–biotin-peroxidase complex in KPBS (ABC Elite; Vector Laboratories; Burlingame, CA, USA). The sections were developed using 3,3-diaminobenzidine and 1% H_2_O_2_. The sections were mounted on gelatine-coated glass slides, dehydrated, cleared in xylene, and cover-slipped with DPX (06,522: Sigma-Aldrich, St. Louis, MO, USA).

*Microscopy analysis* MHCII^+^ cells and cellular structures containing MJF14^+^ misfolded α-Syn were quantified at seven coronal sections of the brains using the 20 × lens of a Leica DMI600B microscope, although a complementary morphological analysis was also run at 40 × of magnification when needed. For CD68, one representative photo of SN at 10 × was taken of each animal; and using Image J, the area covered by immunostaining was calculated for each side and animal.

We performed unbiased stereological quantification of TH^+^ cell bodies in SN based on the optical fractionator principle with an accepted error coefficient of < 0.10, as described previously [46]. Briefly, stereological counting was performed in sections covering the full extent of SN from the rostral tip of the pars compacta to the caudal pars reticulata (approx. 8–9 sections) using the software NEWcast, Visiofarm (vs. 2.14.11.00). A low-power objective lens (1.25x, SPlan) was used to outline the borders of the area of interest in SN. The actual counting was performed with an objective at a magnification of 40x, and the step length was changed to achieve at least 100 cells counted.

*Statistical analysis* All statistical analyses were performed using Prism 7 (Graph Pad software). When appropriate, two-way ANOVA analyses of variance were used to test the interaction between two factors followed by Tukey’s post hoc. Comparison of groups was done by one-way ANOVA followed by Tukey’s multiple comparison test. Paired analysis was applied for repeated measurements and data originating from the same experimental subject. Values of *p < 0.05 were considered to be significant. Outliers were removed by Grubb’s tests (alpha = 0.05). All data were presented as mean ± standard error of the mean (SEM).

### Förster resonance energy transfer (FRET) analysis of aggregated α-Syn

Aggregated α-Syn in mouse brain homogenates was measured with a Förster Resonance Energy Transfer (FRET)-based human α-Syn aggregation kit (Cisbio Perkin Elmer, 6FASYPEH) according to the vendor’s instructions. Aggregated α-Syn was detected using one specific monoclonal antibody, labelled either with Tb-Cryptate (donor) or with d2 (acceptor). When the dyes are in close proximity, the excitation of the donor with a 337 nm light source triggers FRET, shifting the emission from 620 to 665 nm. Signal intensity is proportional to the number of aggregates in the sample. Briefly, 10% brain homogenate in PBS was diluted in a twofold dilution series with cisbio lysis buffer, and all dilutions were mixed with Anti-h-α-Syn-d2 antibody and Anti-h-α-Syn-Tb-Cryptate antibody in three technical replicates in 384-well plates. After 20 h incubation at 18–22 °C, fluorescence emission was read at 665 nm and 620 nm in PHERAstar micro-plate reader. The 665/620 ratio for each individual well corresponds to the amount of aggregated α-Syn. The 64-fold dilution point was chosen as it was found to be in the linear range of detection. This avoids a hook effect, which is seen at more concentrated dilutions in some homogenates, because too high aggregate concentrations capture all antibodies, leading to a plateau and a decrease in signal. The level of aggregated α-Syn in the samples is expressed as delta F%/ug, which is the percentage of “sample FRET ratio *10,000” compared to the “assay-negative control FRET ratio *10,000” normalised to the protein concentration of the homogenate.

### α-Syn protein misfolding cyclic amplification (α-Syn-PMCA) from human A53T-α-Syn-transgenic M83 mice

The PMCA assay was performed essentially as described [92]. In brief, seed-free C-terminally hexa-histidine-tagged human α-Syn (1 mg/ml) in aggregation buffer (100 mM PIPES pH 6.5, 500 mM NaCl) containing 5 μM of thioflavin T (ThT) was placed in an opaque 96-well plate at a final volume of 200 µl and incubated in the presence of brain homogenates from vehicle (PBS), α-Syn PFF, or α-Syn/p25α PFF IM-injected mice (at final concentrations of 0.01, 0.001, and 0.0001%). Samples were subjected to cyclic agitation (1 min at 500 r.p.m. followed by 29 min of no shaking) at 37 °C. The increase in ThT fluorescence was monitored at the *λ*_ex_ = 435 nm and *λ*_em_ = 485 nm, periodically, using a micro-plate spectrofluorometer Gemini-XS (Molecular Devices, Sunnywale, CA).

* Proteinase K digestion of PMCA-amplified aggregates* PMCA-amplified α-Syn aggregates were treated with proteinase K (1 mg/ml) for 2 h at 37 °C. The reaction was stopped by heating the samples in NuPAGE LDS sample buffer at 95 °C for 10 min. The digested samples were resolved by NuPage 12% Bis–Tris gel (Invitrogen). Proteins were electrophoretically transferred to a nitrocellulose membrane (Amersham Biosciences). The membrane was blocked with 5% w/v nonfat dry milk in phosphate-buffered saline-Tween-20 [PBS, 0.1% (v/v) Tween-20] at room temperature for 1 h. After that, the membrane was probed with Syn-1 antibody (BD Biosciences 610787) (1:5000). The blot was developed using ECL Prime Detection Western Blotting Reagents (Amersham Biosciences).

*Preparation of brain homogenates* Frozen brain samples were homogenized in 1X PBS-containing protease inhibitors and phosphatase inhibitors at a final concentration of 10% using Precellys Lysing Kit (Catalogue no. P000912-LYSK0) in a bead homogenizer at 5000 r.p.m. for 15 s. The brain homogenates were aliquoted and stored at − 80 °C until analysis.

## Results

### Structural characterisation of p25α-induced α-Syn fibrils

**Fig. 1:**
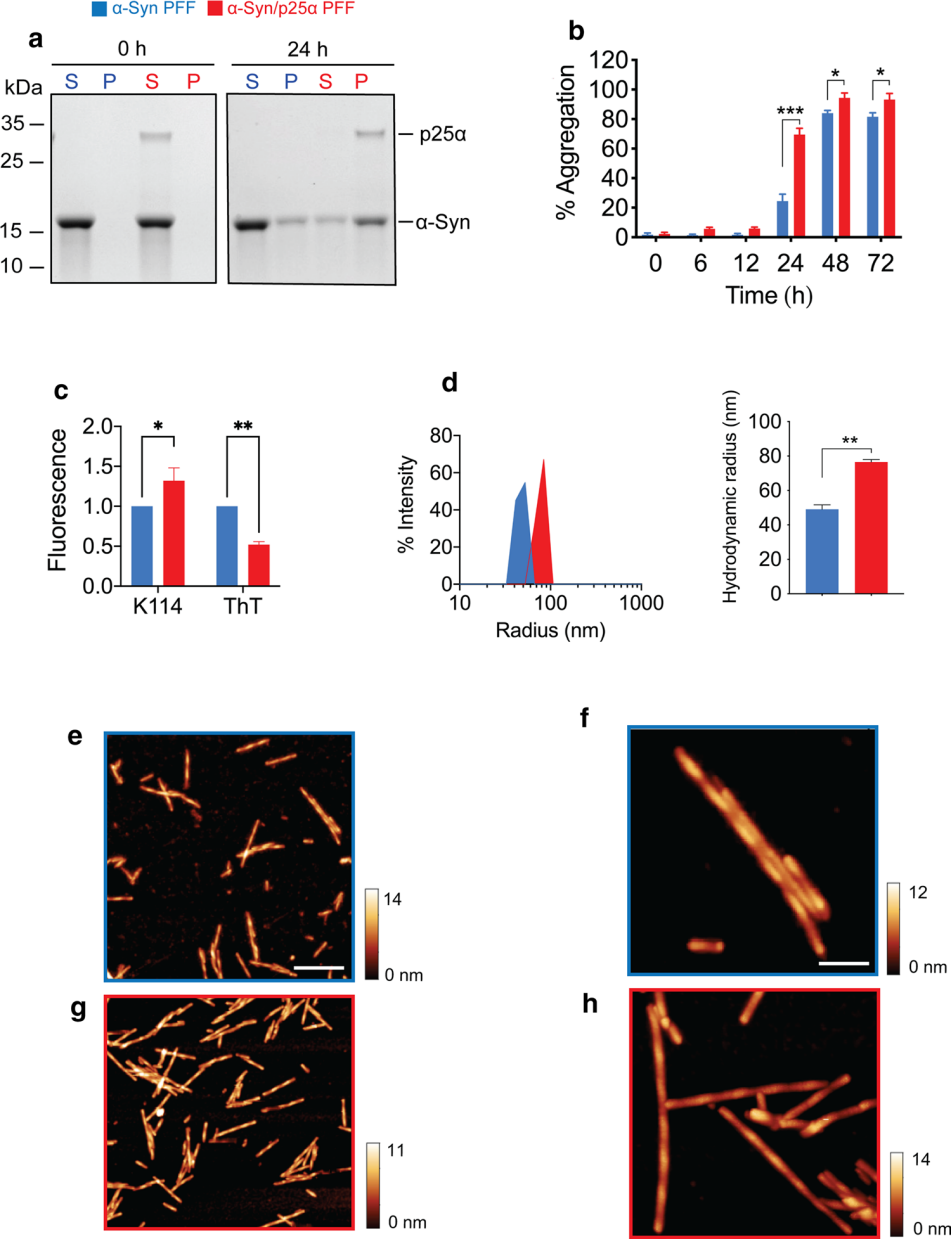
p25α accelerates α-Syn aggregation and binds to α-Syn aggregates. Soluble monomeric α-Syn (346 μM) was assembled in the absence (labelled blue) and presence of p25α (17 μM) (labelled red) into PFF by incubation at 37 °C with continuous shaking for up to  72 h. During the incubation, samples were removed and analysed for solubility by centrifugation and amyloid formation by K114 and ThT fluorescence. **a** Representative Coomassie Blue stained SDS–polyacrylamide gel of supernatant (S) and pellet (P) fractions at the beginning of incubation and after 24 h. **b** Time course for development of insolubility based on densitometric analysis of gels as in panel a. *Y*-axis demonstrates the percentage of aggregation as calculated by [P/(P + S)] × 100]. Bars represent as mean ± SEM of four experiments. *P < 0.05; ***P < 0.001 based on two-way ANOVA followed by Tukey’s multiple comparisons test. **c** K114 and ThT fluorescence of α-Syn and α-Syn/p25α PFF measured at end-stage plateau of aggregation experiment. Y-axis represents arbitrary units normalised to the fluorescence of the control α-Syn fibrils. Bars represented as mean ± SEM of three experiments normalized to α-Syn signal. *P < 0.05; **P < 0.01. **d** Dynamic light scattering (DLS) analysis of α-Syn and α-Syn/p25α PFF aggregates after sonication to shear the filaments. Left panel demonstrates the scattering intensity on the *Y*-axis and the hydrodynamic radius on the log scaled *X*-axis. The right panel displays the hydrodynamic radius of the two fibril populations displayed as mean ± SEM of four experiments. **P < 0.01 based on two-tailed paired *t* test. **e–h** Atomic force microscopy (AFM) characterisation of α-Syn fibrils (**e**, **f**, outlined in blue) and α-Syn/p25α fibrils (**g**, **h**, outlined in red). Scale bar in e = 500 nm also applies to **g** and scale bar in f =  100 nm also applies to **h**

We hypothesised that the encounter between p25α and α-Syn in oligodendrocytes and neurons in MSA leads to the formation of a structurally distinct and toxic strain of p25α-induced α-Syn fibrils, potentially explaining the rapid neurodegeneration in this disease. To test our hypothesis, we first validated the p25α-stimulated α-Syn aggregation kinetics in vitro by comparing the aggregation of soluble monomeric α-Syn in the absence or presence of substoichiometric amounts of p25α [58]. For monitoring α-Syn aggregation, we used a α-Syn sedimentation assay (Fig. 1a, b) and thioflavin T (ThT) and K114 amyloid fluorometry (Fig. 1c) [111]. Using sedimentation analysis, we show that in the presence of 5% p25α as molar ratio, α-Syn aggregates faster than α-Syn alone with 70% compared to 25% α-Syn being insoluble after 24 h (Fig. 1a, b). Both α-Syn strains reached a plateau phase at 48 h, indicating that fibrillation does not proceed further beyond this stage (Fig. 1b). However, the α-Syn/p25α PFF strain comprised a significantly larger fraction of insoluble α-Syn than the α-Syn PFF strain (94% vs 84%). Thus, the presence of p25α decreases the amount of soluble α-Syn co-existing with fibrils. Once fibrillation has reached a plateau level, it is generally understood that an equilibrium has been established between soluble (monomeric) and insoluble (fibrillated) protein. This equilibrium can be modelled as a simple binding reaction between the monomeric protein and the growing ends of the fibril [7, 72]. Thus, the reduction in soluble α-Syn by α-Syn/p25α PFF aggregates indicates that the equilibrium has been displaced towards the fibrillated state compared to the α-Syn PFF aggregates.

At the 24 h time point, all p25α was found in the insoluble fraction (Fig. 1a) despite approximately 30% α-Syn remaining soluble. The specificity of the interaction of p25α with the insoluble α-Syn was validated by comparing the insoluble fractions of α-Syn when aggregated in the presence of p25α and the control proteins carbonic anhydrase (CA) and bovine serum albumin (BSA) (Supplementary Fig. 1a, online resource). Here, it is evident that p25α is associated with the insoluble α-Syn in the pellet fractions in contrast to CA, and BSA remains in the soluble fraction. This highlights how p25α preferentially interacts with aggregated α-Syn fibrils, as previously reported [58].

To validate further the specificity of the p25α-induced α-Syn aggregation, we compared the aggregation kinetics of α-Syn in the presence of p25α and the control proteins CA and BSA. P25α significantly reduced the lag phase of the aggregation, whereas in the presence of CA and BSA, the lag phase was indistinguishable from aggregation of α-Syn alone, as determine by ThT and K114 fluorescence (Supplementary Fig. 1b, c, online resource).

The insoluble aggregates collected after reaching the plateau phase in Fig. 1b consisted of pure and intact α-Syn (Supplementary Fig. 2a, online resource), and their morphologies were analysed by negative-staining transmission electron microscopic (TEM, Supplementary Fig. 2b, online resource) and atomic force microscopy (AFM, Fig. 1e–h). Both α-Syn and α-Syn/p25α PFF presented as twisted, paired, helical filaments, which did not display evident morphological differences to those reported for fibrils [10].

Sonication shears amyloid fibrils into smaller fragments and can be used to assess amyloid nanomechanical properties [1]. Dynamic light scattering (DLS) analysis showed that prolonged sonication of α-Syn and α-Syn/p25α PFF strains resulted in particle populations of different size distributions. These populations displayed hydrodynamic radii of 49.1nm (α-Syn) vs  76.5 nm (α-Syn/p25α) (Fig. 1d). The ability of α-Syn/p25α PFF to withstand ultrasound breakage better than α-Syn PFF suggests a reduced structural rigidity.

**Fig. 2:**
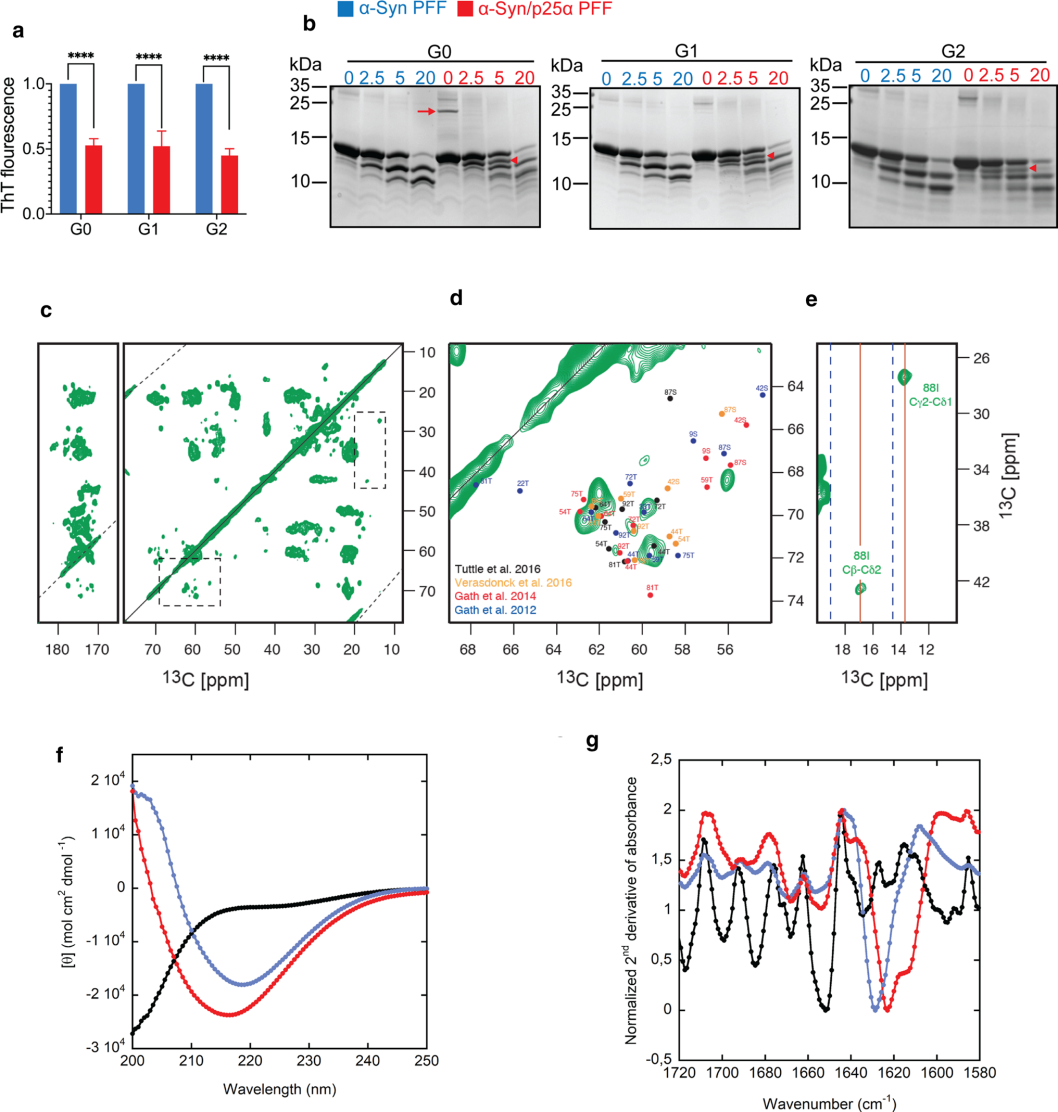
α-Syn strains show different structural organisation. De novo generated α-Syn and α-Syn/p25α parental PFF (G0) were used to seed second- and third-generation fibrils (G1-G2) by seeding of 69 µM α-Syn monomers with 3.5 µM parental α-Syn or α-Syn/p25α PFF as seeds. **a** ThT fluorescence of G0-G2 of the α-Syn and α-Syn/p25α PFF (blue and red, respectively. Values are normalized to the ThT fluorescence signal of α-Syn PFF from the same generation. Asterisks indicate ****P < 0.0001 based on two-way ANOVA followed by Sidak's multiple comparisons test. **b** α-Syn and α-Syn/p25α PFF (blue and red, respectively) incubated with increasing concentrations of proteinase K, indicated in μg/ml above the gels, and resolved by SDS-PAGE followed and Coomassie Blue staining. Molecular size standards in kDa are indicated to the left of each gel. The p25α present in the parental α-Syn/p25α G0 strain is indicated by red arrow and a prominent α-Syn/p25α strain-specific proteinase K fragment that is templated from G0 to G2 is indicated by red arrow head. **c** Solid-state NMR analysis of α-Syn/p25α PFF. **d**, **e** Represents the regions marked with dashed line in panel c. Published chemical shift sets from different structures are inserted in different colours: orange [107], black [103], red [28] and blue [29] presented in **d**. **f** Circular dichroism (CD) spectra and **g** Fourier-transformed infrared spectroscopy (FTIR) spectra of 0.4 mg/ml α-Syn monomer (black line), α-Syn PFF (blue line) or α-Syn/p25α PFF (red line)

The fluorescent dye ThT is used extensively to demonstrate the presence of parallel β-sheet structure in amyloids, and recent evidence demonstrate that its fluorescence can be used to distinguish between α-Syn amyloid fibrils amplified by protein misfolding cyclic amplification (PMCA) from CSF from PD and MSA patients [92] where the MSA-derived fibrils emit less fluorescence. Figure 1c demonstrates that α-Syn/p25α PFF emits approximately 50% of the ThT signal when compared to the control α-Syn PFF. This is in contrast with the emission from the PFF-bound K114 dye, which is significantly higher when bound to the α-Syn/p25α PFF. The first-generation α-Syn/p25α strain (G0) induced by 5% p25α was propagated in monomeric α-Syn by seeding with 5% α-Syn/p25α PFF for two consecutive generations (G1, G2). This dilution resulted in a G2 α-Syn/p25α strain essentially devoid of p25α, but with a retained lower ThT fluorescence than the control α-Syn PFF (Fig. 2a). Thus, our results indicate that the α-Syn/p25α strain presents a different packing of its parallel β-pleated sheet structure, which is propagated in the absence of any p25α molecules.

Limited proteolysis of amyloid fibrils is a useful technique to distinguish between α-Syn strains [10] and proteinase K treatment of α-Syn fibrils and α-Syn/p25α PFF resulted in different proteolytic patterns (Fig. 2b). Interestingly, α-Syn/p25α PFF digested with 2.5–5 µg/ml proteinase K displayed an additional fragment 1 kDa below the band corresponding to the intact protein (arrowhead in red, Fig. 2b) that was nearly absent in the α-Syn PFF samples. Apart from this band, the proteinase-K-treated α-Syn/p25α PFF also displayed a series of smaller molecular weight bands upon proteolysis by 20 µg/ml proteinase K that were absent in the α-Syn fibrils. The limited proteolysis allowed us to demonstrate that the conformational properties of α-Syn/p25α PFF responsible for the distinct cleavage pattern can be templated into daughter generations in the absence of p25α analogous to the low ThT fluorescence (Fig. 2a), as determined by Coomassie Blue staining (Fig. 2b) and LB509 antibody binding (Supplementary Fig. 2c, online resource). These findings demonstrate that α-Syn/p25α fibrils can imprint their conformation in newly formed fibrils in the absence of p25α.

To investigate further the ultrastructural architecture of this new α-Syn/p25α polymorph, we generated fibrils from ^13^C-^15^ N-labelled α-Syn followed by solid-state nuclear magnetic resonance (NMR) analysis. The results showed highly ordered and homogenous fibrils, as reflected by sharp and narrow cross-peaks in the 2D ^13^C-^13^C PDSD spectrum (Fig. 2c–e). A qualitative comparison of the threonine and serine region in the α-Syn/p25α PFF and previously published α-Syn polymorphs [28, 29, 103, 107] (Fig. 2d) indicates that α-Syn/p25α PFF displays a unique amino acid cross-peak pattern that is not fully fitting the hitherto reported α-Syn polymorphs. The corresponding chemical shift assignments previously reported, including the so-called “fibril” and “ribbon”, are indicated in the spectrum with colour coding in Fig. 2d [28, 29, 103, 107] (summarised in Supplementary Table 1, online resource).

A closer look at the threonine cross-peak region (Fig. 2d) reveals eight cross-peaks that can also be identified in the carbonyl region and NCa spectrum (data not shown). This number nicely corresponds to the number of threonine residues in the fibrillar region. The chemical shift values for the isoleucine, I88, Cβ-Cγ2, and Cγ2-Cδ1 cross-peaks shown in Fig. 2e suggest that the α-Syn/p25α PFF resembles the “fibril” conformation rather than the “ribbon” conformation (the corresponding chemical shift values for 88I in “ribbon” and “fibril” conformations are shown as dashed blue and solid red lines, respectively) [28, 29]. As a result, we hypothesise that in the presence of p25α, α-Syn aggregation results in a polymorph varying from previously reported isoforms and more similar to the so-called fibril than the ribbon-type polymorph [10].

Far-UV circular dichroism (CD) (Fig. 2f) and attenuated total reflectance Fourier-transform infrared spectroscopy (ATR-FTIR) (Fig. 2g) were used to evaluate the secondary structure of the α-Syn strains. The results indicated that although both α-Syn polymorphs show CD and FTIR spectra characteristic of parallel β-sheet structure, α-Syn/p25α PFF seems more bona fide amyloid. In FTIR spectroscopy, β-sheets in amyloid fibrils tend to give rise to peaks around 1615–1630 cm^−1^, while β-sheets in globular proteins cluster around 1630–1640 cm^−1^ [117]. The lower the wavenumber, the more “archetypic” the amyloid. α-Syn/p25α PFF showed a minimum around 1623 cm^−1^, significantly lower than the 1628 cm^−1^ minimum of α-Syn PFF. Further support comes from the observation that the minimum of the CD spectrum was shifted to lower wavelengths for α-Syn/p25α PFF (216 nm) than PFF (219 nm); canonical parallel β-sheets typically have a minimum around 217 nm [68].

The surface of α-Syn polymorphs has been probed by aggregate-specific FILA-1 antibody, which recognises conformation-specific epitopes in non-denaturing immuno-dot blot analysis [53, 106] (Supplementary Fig. 2d, online resource). α-Syn antibody Syn-1 was used to assure equal loading of the two strains and demonstrated equal binding to the G0 and G1 generation, and p25α was detectable only in the G0 generation of α-Syn/p25α PFF, as expected. The folding of the two strains resulted in an about twofold stronger binding of the polyclonal aggregate-specific FILA-1 [57] to α-Syn/p25α PFF and an enhanced binding of the monoclonal aggregate-specific MJF-14 [53] was also noted (Supplementary Fig. 2d, online resource).

Overall, our results demonstrate that substoichiometric amounts of the oligodendroglial protein p25α can drive polymerization of α-Syn monomers into a novel fibril strain that can be propagated in new generations without the presence of p25α.

### Seeding of α-Syn aggregation in human iPSCs-derived dopaminergic neurons by α-Syn/p25α fibrils

**Fig. 3:**
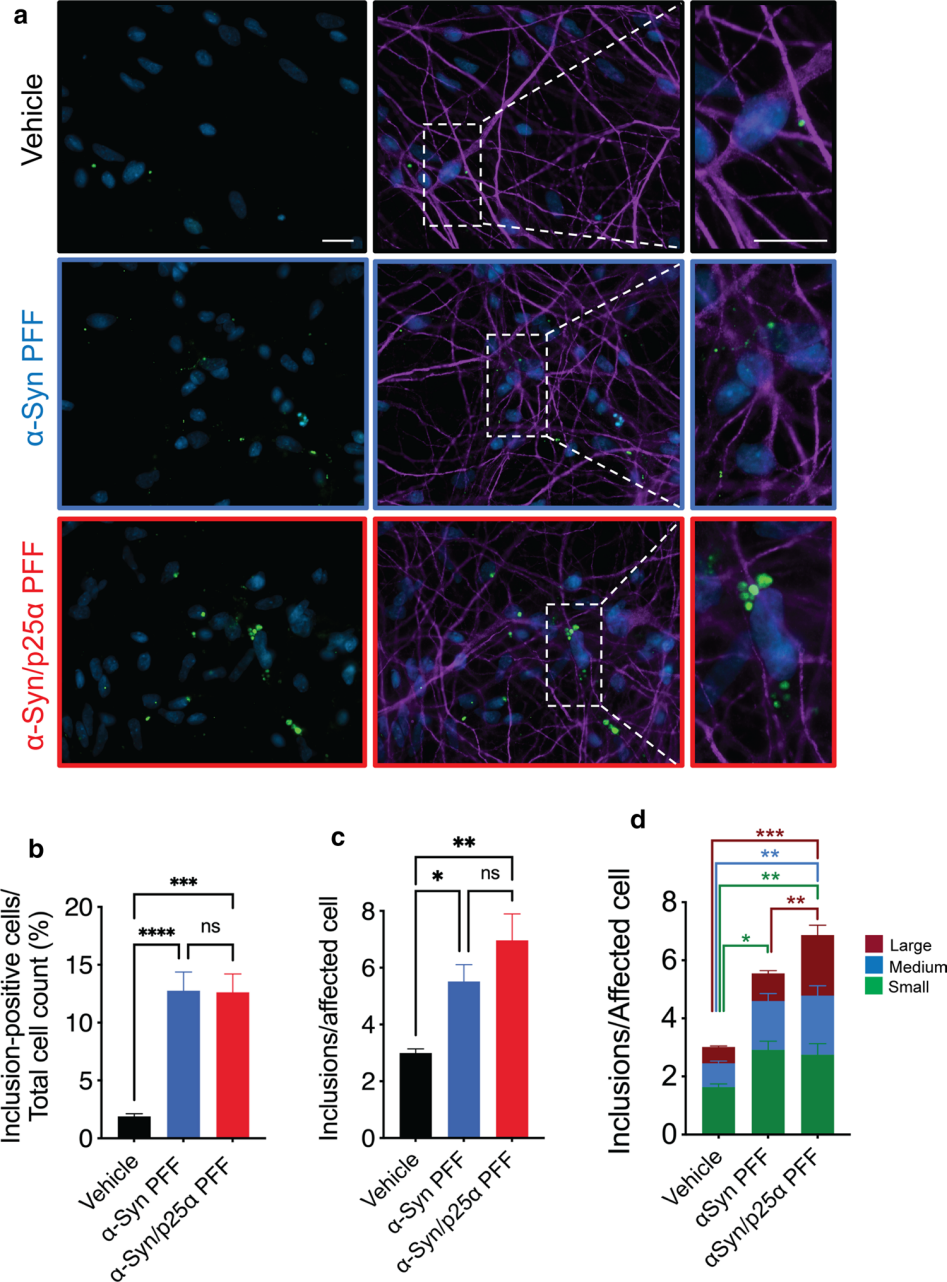
α-Syn/p25α PFF template more and larger α-Syn inclusions in human iPSCs-derived neurons. **a** Human iPSCs-derived neurons were differentiated from neuronal stem cells for 45 days. After 38 days of differentiation, neurons were supplemented with vehicle (upper panels) or G1 generation of α-Syn PFF (middle panels, outlined in blue) and α-Syn/p25α PFF (lower panels, outlined in red) (14 µg/ml) for 24 h followed by media shift to remove excess PFF. They were kept in culture for an additional 7 days in fresh medium before fixation in 4% PFA. Cells were immunostained for microtubule-associated protein 2 (MAP2) (purple) to label neuronal cell bodies and pS129 α-Syn (green) to label inclusions, and the nuclei were stained with DAPI (blue). Pictures were taken randomly with a X63 objective on a Zeiss Observer Z1 microscope. Scalebar = 20 µm. **b** Columns demonstrate percentage of total MAP2-positive neurons carrying inclusions. **c** Columns demonstrate number of pS129-α-Syn-positive inclusions/MAP2 neuron carrying inclusions. **d** Bar graph illustrating inclusion size distribution categorized into small (25–100 pixel^2^, green), medium (100–300 pixel^2^, blue), and large (> 300 pixel^2^, maroon) inclusions for the cells analysed in **c**. In the two differentiations performed, a total of *n* = 308 inclusion-bearing cells were analysed for the α-Syn/p25α PFF group, *n* = 267 cells for the α-Syn PFF group and *n* = 189 cells for the untreated group. Bars represent mean ± SEM from six different cultures derived from two independent differentiations. *P < 0.05, **P < 0.01, ***P < 0.001 based on one-way ANOVA

To investigate the templated cellular seeding of the two α-Syn strains, we differentiated human neural stem cells into dopaminergic neurons using a 45-day protocol [9, 73]. After 38 days of differentiation, neurons were treated with vehicle, α-Syn PFF, or p25α PFF (14 µg/ml) in cell media for 24 h to allow uptake of extracellular PFF and intracellular templating. An incubation period of 24 h was chosen to mimic a “pulse chase” analysis, where a cohort of seeds will initiate a templated aggregation that subsequently deposits in the following 6 days as Ser129-α-Syn-positive intracellular inclusions. Excess of exogenous seeds was washed out, and cells were allowed to grow for additional 6 days in fresh medium (Fig. 3). For all cellular experiments, PFF were made using the mutant S129A, which is nonphosphorylatable at position 129, to allow us to use phosphor-Ser129 as a proxy for aggregation of endogenous intracellular α-Syn. The S129A mutant did not affect the p25α-dependent folding of the α-Syn/p25α strain as determined by proteinase K cleavage analysis (Supplementary Fig. 3a, online resource).

Our results show that although both strains templated the formation of phosphor-Ser129-α-Syn-positive intracellular inclusions in a similar fraction for the cells (Fig. 3a–c), uptake of α-Syn/p25α seeds induced quantitative changes to the size distribution of inclusions with a significant expansion of especially the population of larger inclusions compared to α-Syn PFF (Fig. 3d; Supplementary Fig. 3b, online resource).

We did parallel studies using a rat oligodendroglial cell line (OLN-AS7) stably expressing human α-Syn that was challenged with 14 µg/ml of the two PFF polymorphs (Supplementary Fig. 3c–g, online resource). These cells were treated for 12 h with the two strains before being washed and supplemented with fresh cell culture media and incubated for further 36 h, because we aimed for a “pulse chase-like” analysis of mitotic cells that divides approximately every 24 h. Immunofluorescence microscopic analysis demonstrated that α-Syn/p25α PFF-treated OLN-AS7 cells developed larger and more numerous pSer129-positive inclusions than cells incubated with α-Syn PFF (Supplementary Fig. 3c–g, online resource), resembling our observations using human neurons (Fig. 3).

To address if the increased intracellular load of aggregate inclusions is due to an enhanced uptake of the α-Syn/p25α strain or may reflect strain-dependent differences in their templating activity, we used wild-type OLN-93 cells. These cells do not express endogenous α-Syn, and this allowed us to study the cellular accumulation of the exogenous α-Syn strains being taken up from the medium. Supplementary Fig. 4a, b (online resource) demonstrates that the total α-Syn associated with the cells accumulated in inclusion-like puncta that covered similar areas of the cells after 0.5 and 24 h of incubation for the two strains. This suggests that the cellular uptake and accumulation of α-Syn and α-Syn/p25α PFF are identical.

Overall, our results demonstrate that once α-Syn/p25α PFF are within neurons, they recruit endogenous α-Syn into larger cytoplasmatic inclusions than the control α-Syn strains. The occurrence of a similar strain-specific handling in a mitotic rat cell-line-expressing human α-Syn suggests that this process does not require cell-type-specific factors, but engages core cellular homeostatic machinery. Elucidating the nature of the specific inclusion compartments of the strains will likely require high-resolution analysis.

### Propagation and neurodegenerative activity of α-Syn/p25α fibrils in vivo

To determine if the α-Syn/p25α strain holds greater neurodegenerative potential in vivo, we used two mouse models. First, we used a transgenic mouse model expressing the mutant human A53T α-Syn controlled by the mouse prion protein promoter (M83 line), and where the α-Syn PFF strains are injected into the hindlimb gastrocnemius muscle [86]. This model allows the study of trans-synaptic spreading of α-Syn aggregate through the neuraxis with a well-defined lethal end point that allows quantitative death curve analysis to be performed. Second, we used wild-type C57BL/6 mice injected with the α-Syn PFF strains in the striatum. This model enables us to determine if the seeding can occur in a model with endogenous α-Syn levels, and if the strain-specific spreading from the striatum displays any preference in targeting projecting neurons located in the substantia nigra compared to, e.g., amygdala. Wild-type C57BL/6 mice express endogenous mouse α-Syn, whereas M83 mice additionally express human A53T-α-Syn driven by the mouse prion promoter. For the sake of consistency, we choose to use human PFF for both the M83 and the wild-type mice, because the effect of p25α on mouse α-Syn-derived aggregates should otherwise have been characterised and compared to the human α-Syn/p25α strain. Still, this calls for caution when comparing data on spreading and cytopathology between the two mouse models.

**Fig. 4:**
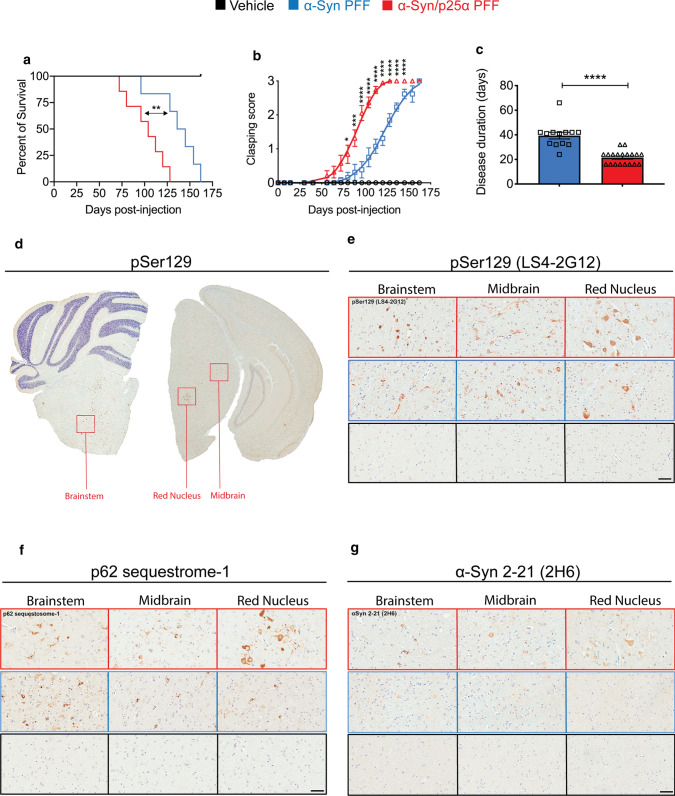
Intramuscular injection of α-Syn/p25α PFF induces earlier and more severe disease than α-Syn PFF in a human A53T α-Syn-transgenic mouse model. Human A53T α-Syn-expressing M83^+/−^ mice were bilaterally injected with α-Syn or α-Syn/p25α PFF (10 μg/leg) or PBS (vehicle control) into the hindlimb gastrocnemius muscle muscle. **a** Kaplan–Meier survival plot shows decreased survival time for age-matched M83^±^ mice injected with α-Syn/p25α PFF (red line, *n* = 19) compared to α-Syn PFF (blue line, *n* = 13) and PBS (black line, *n* = 7). **P < 0.01 as determined by log-rank (Mantel-Cox) test. **b** Hindlimb clasping was scored on a scale from 0 to 3 as a function of days after injection in the mice displayed in panel a and displayed as mean ± s.e.m. The PBS-injected control mice did not develop clasping. Asterisks indicate *P* < 0.05. ***P < 0.001. ****P < 0.0001 based on two-way ANOVA followed by Tukey's multiple comparisons test. **c** Duration of disease (from onset of clasping behaviour until death) for individual mice displayed in panel a injected with α-Syn/p25α PFF and α-Syn PFF. PBS-injected control mice did not develop disease. Error bars indicate mean ± SEM and ****P < 0.001 as determined by one-way ANOVA test. **d** Low-magnification coronal section of a α-Syn/p25α PFF-injected mouse stained with a pSer129 α-Syn-directed antibody LS4-2G12. Boxes indicate locations of brainstem, midbrain, and red nucleus regions analysed in age-matched M83^±^ mice injected with α-Syn/p25α PFF (*n* = 7), α-Syn PFF (*n* = 7), and PBS (*n* = 7). Representative images are displayed of tissue immunohistochemically stained with antibodies reactive to: **e** pSer129 α-Syn (LS4-2G12), **f** p62/sequestosome-1 staining as a marker of general inclusions, and **g** N-terminal of α-Syn using the 2H6 monoclonal antibody against α-Syn 2–21. Scale bar = 50 µm applies to panels e–g

Heterozygous M83 mice (M83^+/−^) were used, because they do not spontaneously develop disease within the time frame of our experimental design if not inoculated with α-Syn PFF. We used the intramuscular (IM) injection of PFF in the hindlimb to induce the lethal disease, because trans-section of the sciatic nerve significantly reduced CNS neuroinvasion and prion-like spread of α-Syn aggregation [86]. Three-month-old M83^+/−^ mice were bilaterally injected in the gastrocnemius muscle with 10 µg of the PFF strains for each injection. The injection needle was inserted 1 mm into the muscle, without damaging the sciatic nerve. Our results demonstrate that compared to α-Syn PFF-injected mice, the α-Syn/p25α PFF decreased median survival time by 26% (Fig. 4a). Using hindlimb clasping as a first sign of detectable disease, we observed that the disease-free period was reduced by 28% (Fig. 4b), and therefore, the disease phenotype progressed 45% faster as determined by time from first clasping to death (Fig. 4c). Other behavioural tests, e.g., grip strength, may have identified a different onset of disease, but would likely not have changed the overall conclusion of a faster disease progression. To identify factors in the brain of affected mice that correlate with the enhanced neurodegenerative phenotype, we compared brains from mice challenged with α-Syn and α-Syn/p25α PFF at the cellular, histological, and biochemical levels.

Our immunohistochemical analysis of coronal sections of the brain focussed on particular areas on the brainstem, midbrain, and red nucleus (Fig. 4d–g; Supplementary Fig. 5–7, online resource). The DAB staining provides semiquantitative information and was especially useful to characterise the presentation of antigens in the cell bodies. The results showed that, as expected, none of the vehicle-injected (PBS) mice displayed any signs of pathology (Fig. 4e–g; Supplementary Fig. 5, online resource). Immunodetection, using a panel of antibodies to investigate differences in α-Syn pathology [15], demonstrated that N-terminal α-Syn-specific antibodies (2H6 in Fig. 4g; and 1D12 in Supplementary Fig. 5f, online resource) preferentially detected pathology in the α-Syn/p25α PFF cohort compared to the control α-Syn PFF mice in all brain regions analysed. Moreover, the 2H6 and 1D12 antibodies specifically stained intracellular inclusions that were more intense and larger in size in the α-Syn/p25α cohort, while they mostly detected pale reactive immunodots in the regular α-Syn PFF mice. Interestingly, the cell bodies of neurons in the red nucleus seem to be particularly targeted by α-Syn/p25α PFF, displaying increased α-Syn epitope immunoreactivity for pSer129 (LS4-2G12 antibody in Fig. 4e; 81A and EP1536Y antibodies in Supplementary Fig. 5e, online resource), α-Syn C-terminal (94-3A10 and 15-4A7 antibodies in Supplementary Fig. 5e, online resource), and α-Syn N-terminal (Syn 506 antibody in Supplementary Fig. 5e, online resource). In addition, a higher number of ubiquitin (Supplementary Fig. 5e, online resource) and p62-positive (Fig. 4f) inclusions were found in the red nucleus of the α-Syn/p25α cohort, indicating increased autophagic and/or proteasomal impairment and neurodegeneration. Quantitative analysis of pixel positivity for the N-terminal antibody 2H6 and the pSer129-directed 81a antibody demonstrated that there was no significant difference in the binding of the two antibodies to the total area of the red nucleus and the brain stem of mice inoculated with both strains (Supplementary Fig. 6, online resource) despite the evidently stronger staining of α-Syn/p25α-challenged cell bodies (Fig. 4e–g). This suggests that the α-Syn/p25α strain induces a stronger formation of inclusions in the cell bodies positive for the two antibodies, analogous to observations in human neurons (Fig. 3), whereas their antigens are more dispersed when induced by the α-Syn strain.

**Fig. 5:**
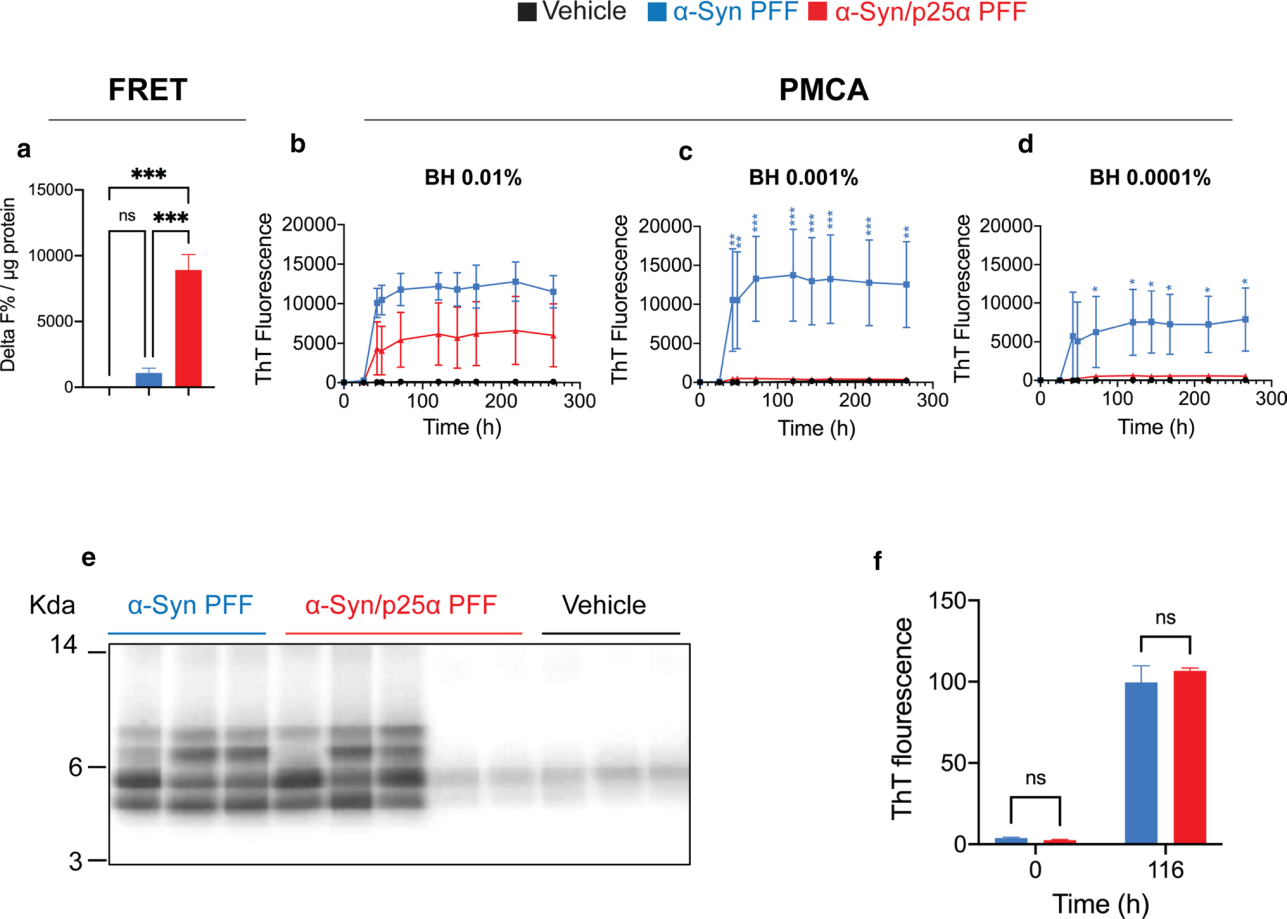
Detection of α-Syn aggregates in mouse brain samples by immuno- and PMCA assay and proteinase K digestion and thioflavin T analyses of amplified aggregates. **a** Brain homogenates of mice injected with α-Syn/p25α PFF contain more aggregated α-Syn than mice injected with α-Syn PFF. Aggregated α-Syn was quantified in brain homogenates using an FRET immune-based Cisbio assay with three technical replicates per homogenate. The level of aggregated α-Syn in the samples is expressed as delta F%/ug protein. This represents the ratio of emission at 665 nm/620 nm of the sample compared to the negative assay control normalized to the protein content (vehicle, *n* = 3, mice #19–21; α-Syn PFF, *n* = 4, mice #3–6; α-Syn/p25α PFF *n* = 4, mice mice #12–15). Neuropathological information of mice samples is available in Supplementary Table 2, online resource. BCA protein measurements determined the total protein concentration of the homogenates. One-way ANOVA followed by Tukey’s post hoc. Bars show mean ± SEM ***P < 0.001. **b-d** Brain homogenates of mice injected with α-Syn/p25α PFF contain less templating active seeds than mice injected with α-Syn PFF. Brain samples from mouse inoculated with α-Syn PFF (blue line, *n* = 3, mice #1–3), α-Syn/p25 PFF (red line, *n* = 5, mice #7–11), and vehicle (PBS, pH 7.4, black line, *n* = 3, mice #16–18) were homogenized at 10% w/v. Brain samples (at final concentrations of 0.01, 0.001 and 0.0001%) were analysed in a 96-well plate α-Syn-PMCA assay. The extent of aggregation was monitored by the increase in ThT fluorescence by a spectrofluorometer using an *λ*_ex_ = 435 nm and an *λ*_em_ = 485 nm. Neuropathological information of mice samples is available in Supplementary Table 2, online resource. The experiments were carried out in duplicates, and error bars indicate mean ± s.e.m. Asterisks indicate *P < 0.05. **P < 0.01. ***P < 0.001 based on two-way ANOVA followed by Tukey's multiple comparisons test. **e** PMCA-amplified α-Syn aggregates (G0) were incubated with proteinase K (1 mg/ml) at 37 °C for 2 h. Proteins were separated on 12% Bis–Tris gel and immunoblotted with anti-alpha synuclein Syn-1 to visualise cleavage patterns. Molecular weight markers are indicated on the left in kilo-Daltons (KDa). **f** G1 of PMCA-amplified α-Syn aggregates were generated by seeding of 69 µM α-Syn monomers with 3.5 µM parental aggregated PMCA-amplified α-Syn material (G0). ThT fluorescence was measured at beginning (0 h) and end-stage plateau (116 h) of the G1 re-amplification experiment. Bars represented as mean of signal from α-Syn PFF-injected mice (blue, *n* = 3) or α-Syn/p25α PFF-injected mice (red, *n* = 3,) ± s.e.m analysed by two-way ANOVA followed by Sidak’s multiple comparisons test. *ns* not significant

To investigate the levels of α-Syn aggregates in the affected tissue, we extracted frozen tissue corresponding to the pons and medulla oblongata (rich in α-Syn pathology in the M83 model). The tissue homogenates were analysed using an FRET immune-based Cisbio platform. Figure 5a demonstrates the FRET signal obtained after 20 h of incubation using a 256-fold dilution of the 10% brain homogenate. The α-Syn/p25α-injected mice (*n* = 4) clearly stand out with a consistent and significantly larger signal than the α-Syn PFF (*n* = 4) and the PBS-control-injected mice (*n* = 3), demonstrating that they contain larger amounts of immuno-reactive α-Syn aggregates (Fig. 5a; Supplementary Fig. 7a, online resource). The α-Syn PFF and the PBS control do not differ significantly, because of one α-Syn PFF-injected outlier that showed very low signal (Supplementary Fig. 7d, online resource). Hence, the α-Syn/p25α cohort that suffers a more severe disease phenotype and displays stronger cell body-associated α-Syn pathology (Fig. 4e–g) harbours larger amounts of α-Syn aggregates than the mice injected with the α-Syn PFF (Fig. 5a; Supplementary Fig. 7a, online resource).

We next asked if the increased histopathology and biochemically detectable aggregate load correlate with an increased in vitro seeding activity that hypothetically could be responsible for the faster disease progression. To quantify the seeding activity of templating active α-Syn aggregates, we used a PMCA technique. This technique can also generate substantial amounts of amplified α-Syn aggregates, which allows subsequent biochemical analysis [92]. Using PMCA, we analysed the same pons/medulla region as used for the Cisbio FRET analysis. Figure 5b demonstrates that a significant ThT signal developed after a lag phase of about 20 h when incubating recombinant HH-tagged-α-Syn with 0.01% homogenates from sick end-stage α-Syn/p25α (*n* = 5) and the α-Syn PFF (*n* = 3) injected mice. By contrast, no signal developed when using healthy controls of similar age (*n* = 3) injected with PBS. Analysis of individual mice revealed that all the end-stage mice injected with α-Syn PFF (*n* = 3) displayed ThT signals that reached a similar plateau of around 10–15,000 AU (Supplementary Fig. 7b, online resource). This contrasted the α-Syn/p25α PFF-injected cohort, where two mice did not seed any aggregation, two generated signals of approximately 15–20,000 AU, and one mouse generated a signal around 1000 (Supplementary Fig. 7b, online resource). Strikingly, when diluting the tissue homogenates tenfold to 0.001% and 0.0001% prior to addition to the PMCA assay, the positive signals from the α-Syn/p25α-injected mice were lost. This contrast with the α-Syn PFF-injected cohort where two mice remained positive at the highest dilution (Fig. 5c, d; Supplementary Fig. 7b-d, online resource). The data demonstrate variation within each small cohort, which was especially pronounced for the α-Syn/p25α-injected mice. Proteinase K digestion of the parental generation 0 (G0) amplified aggregates demonstrated similar Syn-1 immuno-reactive band patterns in α-Syn and α-Syn/p25α cohorts, while a strong intragroup variation was observed in both cohorts (Fig. 5e). The band patterns did not allow the α-Syn/p25α and α-Syn groups to be distinguished. The parental G0 samples contained both monomer HH-α-Syn and diluted brain homogenate, so we reamplified the G0 samples in recombinant non-HH-tagged α-Syn to G1 and G2 samples using 5% insoluble aggregates as seeds. Using this approach, we were able to demonstrate that aggregates amplified from the two α-Syn strain-injected groups were indistinguishable with respect to maximal ThT signals reached in the plateau phase (Fig. 5f). Hence, neither the activity of α-Syn templating-competent seeds in the tissue nor the PK-digestion pattern of the templated aggregates allowed us to distinguish between the two groups injected with different α-Syn strains.

**Fig. 6:**
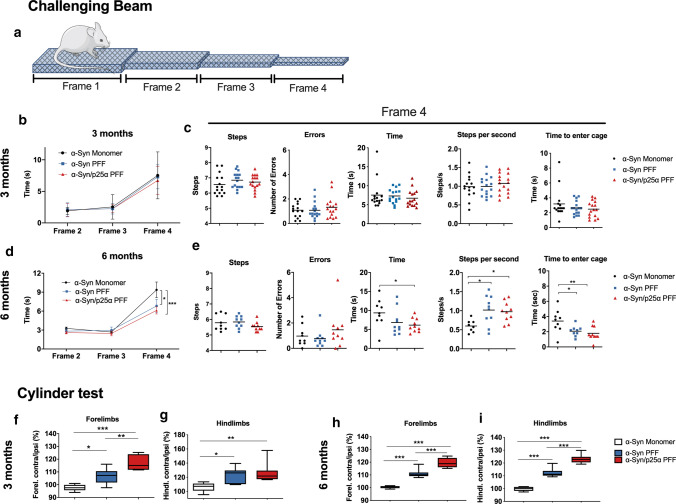
α-Syn/p25α PFF induces abnormal, progressive motor hyperactivity in wild-type mice. Wild-type mice were injected with α-Syn or α-Syn/p25α PFF (10 μg) or monomer α-Syn (10 μg) as negative control in their right striatum and were analysed for motor function by the challenging beam and cylinder test at 3 and 6 months after injection. **a** The challenging beam test measures the time spent, steps taken, and errors in crossing a progressively thinner beam and to enter a cage after the last frame 4. **b-c** No significant changes were observed between the three groups at 3 months after injection (α-Syn monomeric, *n* = 15; α-Syn PFF, *n* = 16; α-Syn/p25α PFF, *n* = 16), **d** 6 months after injection, both α-Syn PFF and α-Syn/p25α PFF-injected animals transversed frame 4 faster than α-Syn monomeric animals (α-Syn monomeric, *n* = 9; α-Syn PFF, *n* = 10; α-Syn/p25α PFF, *n* = 10). Two-way ANOVA followed by Tukey’s post hoc. Error bars indicate mean ± SEM. *P < 0.05. ***P < 0.001. **e** The faster crossing of frame 4 for both groups was due to more steps/s than the α-Syn monomeric animals and they also used less time to enter the home cage. One-way ANOVA followed by Tukey’s post hoc. Error bars indicate mean ± SEM. *P < 0.05. **P < 0.01. The cylinder test revealed an early and persistent side-bias hyperactivity of PFF-injected animals that at 3 month **f**–**g** where animals injected with both PFF polymorphs used the contralateral limbs more than α-Syn monomeric-injected mice, and with the α-Syn/p25α PFF even more strongly affecting the forelimb than in the α-Syn PFF mice (α-Syn monomeric, *n* = 6; α-Syn PFF, *n* = 8; α-Syn/p25α PFF, *n* = 8). One-way ANOVA followed by Tukey’s post hoc. Error bars indicate mean ± s.e.m. *P < 0.05. **P < 0.01. ***P < 0.001. **h**, **i** This difference between the α-Syn PFF and the α-Syn/p25α PFF mice became more obvious at 6 months after injection, where there also was a difference in hindlimb use. (α-Syn monomeric, *n* = 9; α-Syn PFF, *n* = 10; α-Syn/p25α PFF, *n* = 10). One-way ANOVA followed by Tukey’s post hoc. Bars show min and max. ***P < 0.001

To determine if the two strains can induce different pathology and behavioural deficits in wild-type female C57BL/6 mice, we performed unilateral stereotaxic intracerebral injections (IC) of the two strains into the striata of wild-type mice and followed the animals at 1, 3, and 6 months after injection. As controls, we injected the mice with similar amounts of monomer α-Syn. The mice were subjected to two behavioural tests, the challenging beam (Fig. 6a–e) and the cylinder test (Fig. 6f–i). In the challenging beam, the mouse has to traverse a beam with four frames that become progressively narrower and to finally enter its cage. At 3 months after injection, none of the cohorts showed any significant difference in motor behaviour in the challenging beam (Fig. 6b, c), while 6 months after injection, both groups of animals injected with α-Syn aggregate strains exhibited hyperactivity compared to the controls as demonstrated by shorter time to cross and more steps per second (Fig. 6d, e). They were also quicker to transverse the last frame and enter the home cage (Fig. 6c, e). In the cylinder test, the use of fore- and hindlimbs was quantified to determine the presence of asymmetry in the use of limbs. As early at 3 months after injection, the mice injected with aggregated α-Syn strains developed asymmetry and used the contralateral fore- and hindlimbs more that the controls (Fig. 6f, g). The motor asymmetry of the forelimbs was significantly higher in the α-Syn/p25α cohort than in the α-Syn PFF mice (Fig. 6f), and it became also apparent in the hindlimbs at 6 months after injection (Fig. 6i). Thus, injection of aggregated α-Syn resulted in a progressive and asymmetric motor hyperactivity that was more-pronounced in the α-Syn/p25α cohort.

**Fig. 7:**
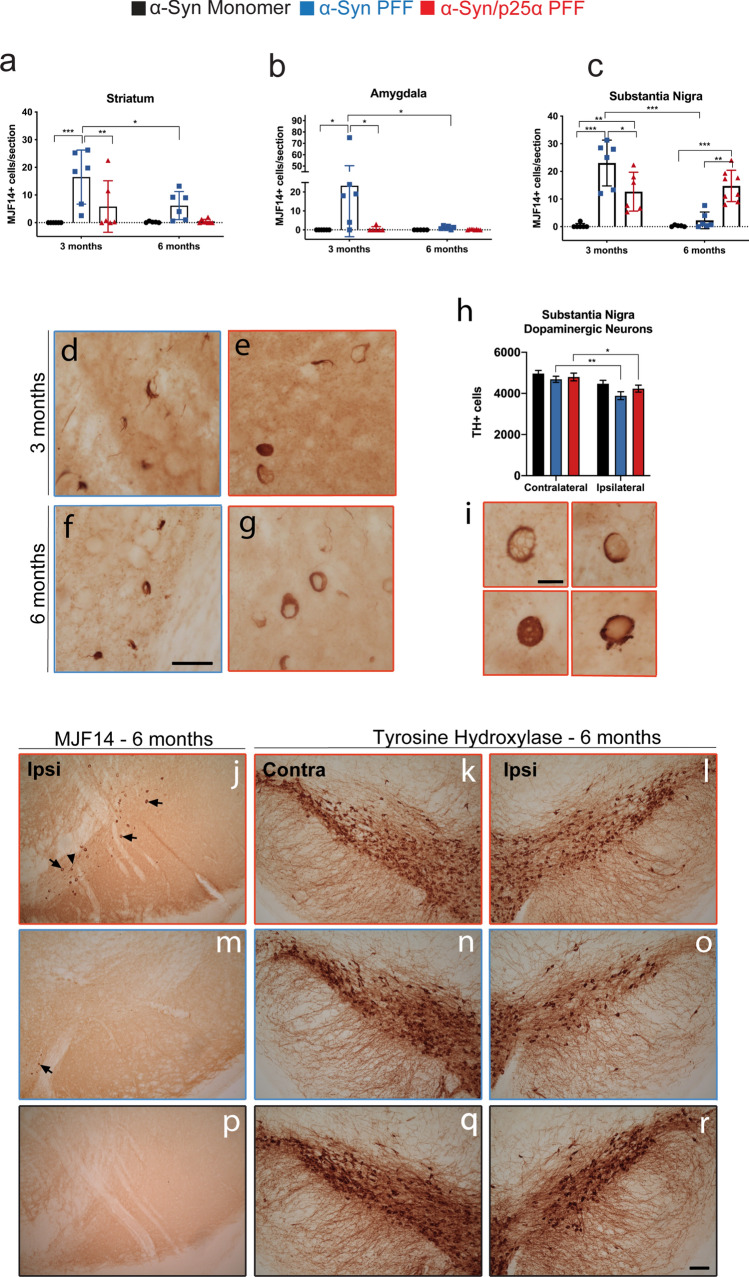
α-Syn/p25α PFF induces preferential α-Syn aggregation in nigro-striatal neurons. Progression of the α-Syn pathology and dopaminergic neurodegeneration in mice injected with α-Syn monomer, α-Syn PFF or α-Syn/p25α PFF was assessed by immunohistochemistry and stereology. **a**–**c** Brain sections were immunostained with an antibody against aggregated α-Syn (MJF-14); the labelled cell body structures were counted in striatum, amygdala, and substantia nigra (SN), and the average number of cells per coronal section in each group was calculated at 3 and 6 months after injection. **d**–**g** Representative images of cellular structures immunostained with MJF-14 antibody in the SN. Notice that MJF-14-labelled structures observed in the ventral midbrain cells of α-Syn PFF-injected animals extended into neurites and preferentially were segregated into parts of the perinuclear space (**d**, **f**); that contrasts with the circular perinuclear staining found in the mice injected with α-Syn/p25α PFF (**e**, **g**). **h** Stereological quantification of tyrosine hydroxylase (TH)-positive cells in SN showed a significant decrease of neurons in the ipsilateral side of the α-Syn PFF-injected group and in the α-Syn/p25α PFF-injected group, while no changes were found after monomeric α-Syn injections. In **i**, details of the different cells found in SN of the α-Syn/p25α PFF animals at 6 months are presented. **j**, **m**, **p** Representative images of SN immunostained with MJF-14 antibody in all three groups at 6 months. Notice that MJF-14^+^ structures observed in the α-Syn PFF-injected animals were normally located in the ventral medial midbrain in the border with VTA (**m**), while cells (arrows in **j**) and fibres (arrowhead in **j**) were located in the SN compacta in the α-Syn/p25α PFF animals. **k**, **l**, **n**, **o**, **q**, **r** Representative images of the contralateral SN and its correspondent ipsilateral side from sections immunostained with TH antibody in all three groups at 6 months. Notice the decrease in the number of cell bodies in the ipsilateral side of the α-Syn PFF-injected mice in **o** and the α-Syn/p25α PFF-injected mice in (**l**). Data are average ± SD. **a**–**c** or SEM. **h** Two-way ANOVA followed by Sidak post hoc. * *P* < 0.05. ** *P* < 0.01. *** *P* < 0.001. Scale in *f* = 25 µm applies to **d**–**g**, scale in *i* = 10 µm and applies to insets and scale in t: 100 µm applies to (**j**–**r**)

Immunohistochemical analysis of the brains showed no obvious α-Syn pathology 1 month after injection of the PFF, as assessed by the MJF-14 antibody (not shown). However, at 3 months, both groups injected with aggregated α-Syn strains showed MJF14^+^ structures in the area of injection (striatum) and connected anatomical regions, while mice injected with monomeric α-Syn showed no apparent positive MJF14 staining (Fig. 7a–c; Supplementary Fig. 8, online resource). Especially animals receiving α-Syn PFF showed obvious pathological α-Syn accumulations in cortex, piriform cortex, amygdala, and SN (Supplementary Fig, 8, online resource). By contrast, the MJF14^+^ structures in the α-Syn/p25α cohort were mostly confined to the striatum and SN, with significantly fewer cellular structures than in α-Syn PFF mice (Fig. 7a–c; Supplementary Fig. 8, online resource). This suggests that the α-Syn PFF strain had an ability to induce progressive histopathology in neurons of multiple brain areas similar to that of the α-Syn/p25α PFF that exhibited a “tropism” for nigro-striatal neurons. The number of MJF14^+^ cell bodies decreased significantly after 3 months in all areas in the α-Syn PFF mice (Fig. 7a–c). However, in the α-Syn/p25α cohort, the number of MJF14^+^ cells in SN remained elevated and, at this point, was significantly higher than in the α-Syn PFF animals (Fig. 7c). The α-Syn PFF-induced MJF14^+^ skein-like structures in the neurons were detectable from 3 month and onward (Fig. 7d, f). Already at 3 months, the α-Syn/p25α strain induced perinuclear ring-like and half-moon-shaped inclusions and even dense staining that covered the entire cell body (Fig. 7e). At 6 months, most of the MFJ14^+^ inclusions appeared perinuclear and were preferentially accumulated in the SN pars compacta but not in striatum (Fig. 7g, j). By contrast, the MJF14^+^ cells in the midbrain of the α-Syn PFF-injected mice were fewer, more granular, and seemed more constrained to the border with the ventral tegmental area while still prominent in striatum (Fig. 7a, f, m). Stereological analysis of the tyrosine hydroxylase (TH^+^) dopaminergic neurons in SN showed a significant decrease of TH^+^ nigral neurons in the ipsilateral side of the midbrain (Fig. 7h), of the α-Syn PFF (Fig. 7n, o) and the α-Syn/p25α PFF mice (Fig. 7k, l). Interestingly, at 6 months after α-Syn/p25α PFF inoculation, the percentage of surviving TH^+^ cells (as compared to contralateral TH^+^ cells) was negatively correlated with the number of MJF14^+^ cells in SN; therefore, animals with a higher number of cells containing aggregated α-Syn showed more dopaminergic loss, while such a correlation was not found in the α-Syn PFF group (Supplementary Fig. 9b, online resource).

Interestingly, we observed that the brains’ inflammatory response was significantly lower in the α-Syn/p25α PFF mice than in the other groups, including monomeric α-Syn (Supplementary Fig. 10, 11, online resource). This was true when both astrocyte and microglia response were analysed. GFAP upregulation was observed in the ipsilateral striatum 1 month after injection of α-Syn. This upregulation was highest in α-Syn PFF, followed by monomeric animals with the α-Syn/p25α PFF-injected animals showing the lowest upregulation (Supplementary Fig. 10, online resource). This astrocytic response was decreased after 6 months in all three groups, at a point where the contralateral striatum showed more GFAP^+^ than at 1 month, suggesting a bi-lateralization of the glia response (Supplementary Fig. 10, online resource). Microgliosis, as revealed by Iba1 immunostaining, was observed in the ipsilateral striatum after 1 month in all three groups; but again, the highest changes in Iba1^+^ cell number and morphology were found in the α-Syn PFF group, while the α-Syn/p25α PFF animals showed the lowest number and morphology (Supplementary Fig. 11, online resource). Confirming the lower neuroinflammatory activation in the α-Syn/p25α animals, while MHCII expression in the ipsilateral striatum was upregulated in all three groups, the neuroinflammatory activation was significantly higher in the α-Syn PFF animals than in the other two groups, with the α-Syn/p25α PFF showing a very scarce MHCII expression (Supplementary Fig. 12a, c–e, online resource). These striatal MHCII-ramified cells disappeared after 3 months in all three groups (Supplementary Fig. 12a, online resource), and no significant MHCII expression was found at 6 months (not shown). Ipsilateral Iba1^+^ microgliosis appeared lower after 6 months, but α-Syn PFF animals showed cells with higher Iba1 expression and numerous ramifications, while this was not observed in α-Syn/p25α PFF mice. The bi-lateralization of the neuroinflammatory response also appeared true for microglia. In the SN, GFAP or Iba-1 expression did not appear different among sides and groups at 1 month (not shown) or at 6 months (Supplementary Fig. 10 and 11, online resource). However, we observed a general bilateral upregulation of the phagocytic marker CD68 in the SN (which normally is expressed at very low levels in the mouse brain) in both monomeric and α-Syn PFF mice. However, we found a significantly lower CD68 expression in the α-Syn/p25α cohort (Supplementary Fig. 12b,fh, online resource), which is in accordance with the lower neuroinflammatory response in this group.

In conclusion, compared to the control α-Syn PFF, α-Syn/p25α PFF induce a faster onset and more aggressive disease phenotype in the human A53T α-Syn-transgenic mouse model. In wild-type mice, striatal inoculation of α-Syn/p25α PFF elicited a certain “tropism” for nigro-striatal neurons, where it induced a greater motoric deficit in the cylinder test and a distinctive long-lasting α-Syn pathology correlating with neuron death; and this occurred on the background of a reduced neuroinflammatory response compared not only to the α-Syn PFF but also to monomeric α-Syn.

## Discussion

Although the three synucleinopathies PD, DLB, and MSA share the progressive development of α-Syn aggregate-containing cytoplasmic inclusions, the histopathology in MSA exhibits a range of special characteristics. The most recognised feature is that the burden of α-Syn inclusion pathology is larger in MSA than in PD and DLB, and the cytoplasmic inclusions predominantly reside in oligodendrocytes [17, 36, 66]. Such oligodendroglial cytoplasmic inclusions also exist, although less abundantly, in PD and DLB associated with familiar α-Syn mutations and in the pallidothalamic tract in sporadic DLB [45, 75]. However, in MSA, α-Syn aggregates also frequently occur in neurons as neuropil threads and in neuronal nuclear and cytoplasmic inclusions [36, 66]. The significant neuronal loss in MSA-affected regions might result in underestimation of the prevalence of α-Syn neuronal inclusion that occurred in the tissue [36, 112]. At the molecular level, the α-Syn molecules in GCI are organised differently from those in LB as demonstrated by different presentation of α-Syn epitopes [19, 65]. These observations were corroborated by the high-resolution structure of two unique α-Syn polymorphs isolated from MSA brains that differed from those isolated from DLB brains [89]. In addition, α-Syn aggregates amplified from CSF samples of MSA patients with the PMCA technique display a lower ThT fluorescence than aggregates amplified from PD patients [92], which is a characteristic shared with our p25α-induced PFF polymorph. Functionally, α-Syn aggregate seeds from MSA brain extracts induce a very aggressive disease phenotype compared to PD when injected into brains of the human A53T-α-Syn-transgenic M83 mouse line [79, 108]. This enhanced toxicity is likely mediated by intrinsic structural characteristics, as it can be recapitulated in cells and rodents upon inoculation of PFF generated in vitro via PMCA of α-Syn seeds isolated from MSA brain extracts [54, 106].

The molecular mechanisms causing α-Syn to aggregate into functionally more aggressive polymorphs in MSA remain a conundrum, but the intracellular oligodendroglial milieu has been hypothesised to be responsible for forming these strains [77].

**Fig. 8:**
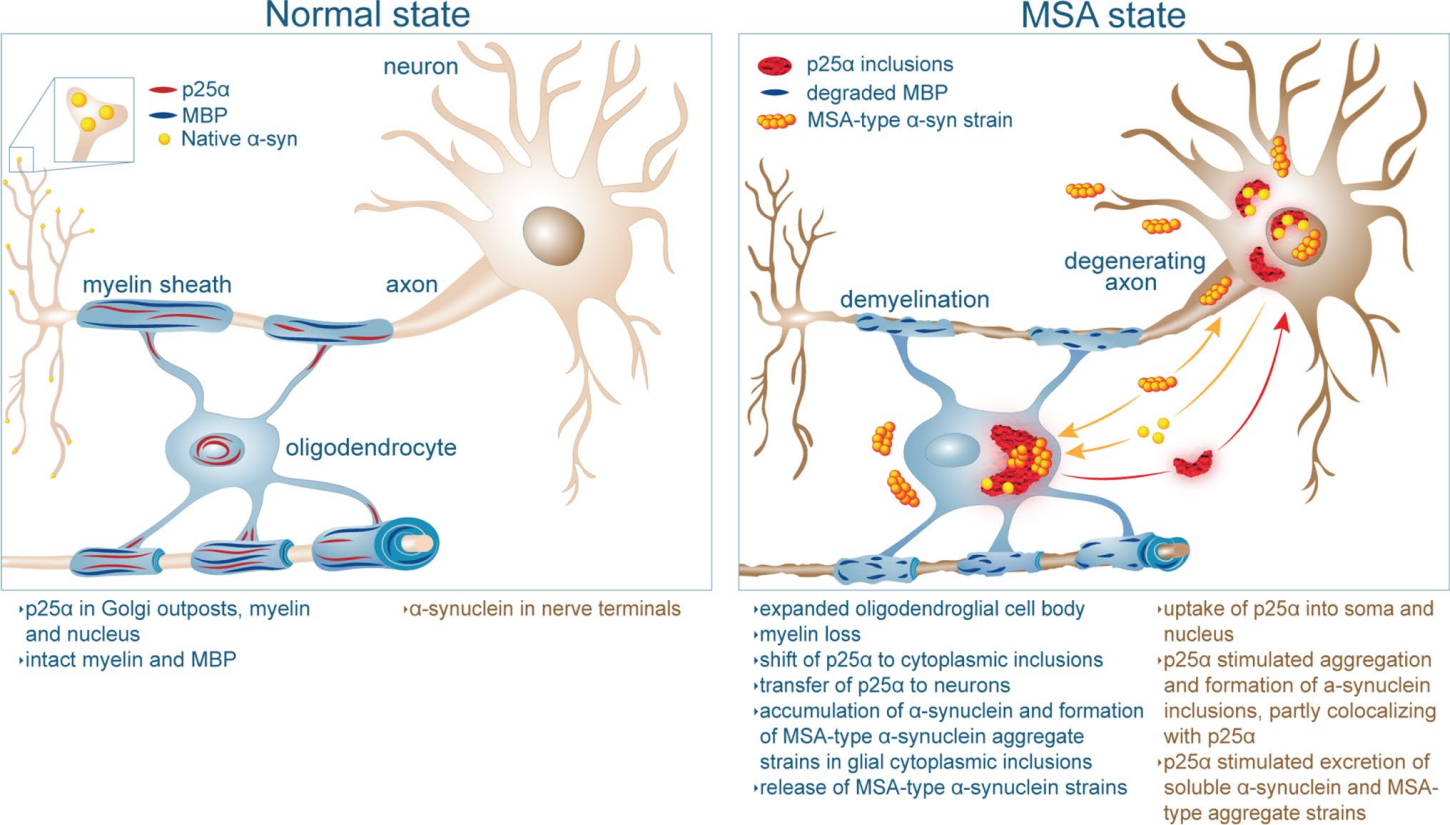
Hypothetical mechanism for the generation of an MSA-associated α-Syn strain with increased neurotoxicity induced by oligodendroglial protein p25α/TPPP. Left panel: p25α/TPPP is expressed in mature oligodendrocytes and is vital for reorganisation and stabilisation myelin. In the physiological state, p25α/TPPP can be found in Golgi outposts in myelin outside the cell body and in the nucleus. The myelin basic protein (MBP) is present in intact myelin. Right panel: In MSA, the oligodendroglial cell body expands, and MBP is degraded leading to demyelination. p25α/TPPP retracts from myelin and exits the nucleus forming early cytoplasmic inclusions. Low levels of α-Syn monomers are then recruited to the inclusion where P25α/TPPP nucleate them into MSA-type α-Syn aggregate strains completing the formation of mature glial cytoplasmic inclusions. P25α/TPPP is released from oligodendrocytes and taken into neurons where it exists in nuclear and extranuclear inclusions in the absence and presence of α-Syn and induce formation of MSA-type α-Syn polymorphs that accumulate in neurites and nuclei as neuronal nuclear and cytoplasmic inclusions causing neuronal degeneration. The P25α/TPPP facilitates the spreading of the disease process by stimulating atypical secretion of α-Syn aggregates

We hypothesised that the oligodendroglial protein p25α is a causative factor that contributes to both the oligodendroglial milieu but also facilitates direct prodegenerative actions in neurons and between neurons and oligodendrocytes (Fig. 8).

Within oligodendrocytes in MSA, p25α redistributes from its normal positions in myelin and the nucleus to accumulate in α-Syn-negative perinuclear inclusions that may represent immature GCI [94]. Here, α-Syn gradually appears and transforms them into mature GCI [48, 58, 94] in a process that parallels the MSA-dependent expansion of the oligodendroglial cell body [74, 83]. The interaction between α-Syn and p25α stimulates α-Syn aggregation [58] and α-Syn aggregate-dependent dysfunctions in p25α-expressing cells including oligodendrocytes (Fig. 8) [21, 51, 65]. P25α may even represent the unknown factor in myelin-rich white matter extracts of α-Syn knockout mice that triggered α-Syn aggregate pathology when injected into the human A53T-α-Syn-transgenic M83 line [85]. The presence of α-Syn in the MSA oligodendrocytes has been the subject of several investigations, because α-Syn is not considered an oligodendroglial protein [71]. However, a recent qPCR analysis of oligodendrocytes isolated from human brain demonstrated α-Syn mRNA in oligodendrocytes [4]. Moreover, oligodendroglial cell lines and in vitro cultured human and rodent oligodendroglial cells express α-Syn mRNA and protein [18, 32, 65] that upon aggregation, e.g., triggered by p25α or templated by exogenous α-Syn seeds, increases the low level of endogenous α-Syn by forming stable α-Syn aggregates [44, 44, 65]. Such aggregation of endogenous α-Syn in oligodendrocyte precursor cells, which remain in the cell through the further differentiation into oligodendrocytes, has been hypothesised to represent a mechanism for the prodegenerative activity of α-Syn in MSA [43]. Moreover, it may be involved in the oligodendroglial down-regulation of myelin genes, neuronal upregulation of synapse genes, and glial inflammatory response observed in MSA-C tissue [78].

P25α also has direct roles in neurons affected by neurodegenerative diseases, because it is present in pretangle-bearing neurons of Alzheimer’s disease [48] and co-exist with α-Syn in LB in PD and DLB. In MSA, p25α was detected in 40% of neuronal cytoplasmic α-Syn inclusions and in 10% of neuronal nuclear α-Syn inclusions and also occurred in inclusions without α-Syn [6]. Hence, while p25α expression in neurons under physiological conditions is considered low, in individual neurons in MSA, it exists in significant levels that may initiate aggregation of α-Syn into aggressive strains. P25α also stimulates unconventional secretion of α-Syn aggregates from neurons by blocking autophagic pathways [21]. This can contribute to non-cell autonomous functions by activation of microglia and astrocytes [98] and transport of templating active α-Syn polymorphs into neighbouring oligodendrocytes and neurons. This could form the basis for the strong neuroinflammation and neuronal loss in MSA.

Our p25α-induced α-Syn strain differed from the α-Syn control strain on several biochemical parameters that have been demonstrated to vary between strains amplified from MSA and PD [92, 106]. This ranged from differential binding of the fluorescent amyloid dye thioflavin T and the aggregate-specific FILA-1 antibody [106] to subtle changes in secondary structure (CD and FTIR) and altered proteolytic peptide patterns as demonstrated by Coomassie Blue staining and immunoblotting with Syn-1 and LB509 antibodies [77, 92, 106].

Higher resolution analysis of the α-Syn/p25α strain by solid-state NMR spectroscopy allowed us to demonstrate that the α-Syn/p25α strain exhibits a novel structure which, based on Ile88, resembled the reported fibril more than the ribbon structure [28, 29]. Our results indicate that induction of α-Syn aggregation by a brain protein or brain extract holds promise for generating more pathophysiological α-Syn polymorphs than those induced by simply changing buffer conditions [102].

We used substoichiometric concentrations of p25α to induce the formation of the α-Syn/p25α polymorph, because our aim was to demonstrate that this oligodendroglial factor, which likely occurs in low amount in neurons, holds potential for inducing a novel α-Syn strain. We validated the specificity of the p25α-induced α-Syn aggregation by showing that control proteins, such as BSA and carbonic anhydrase, neither enhanced aggregation nor induced formation of a novel folding, as determined by fluorometry. The α-Syn/p25α strain can be sustainedly seeded and templated over multiple generations in vitro, using pure preparations of α-Syn monomers, in the absence of p25α. This assures that their functional properties do not rely on any p25α molecules associated with the fibrils, but rely on the actual folding of the fibrils. In this paradigm, p25α acts at the level of building the seed, but is not as such an inherent part of the final α-Syn strain. The initial induction of α-Syn aggregation by p25α may occur in both neurons and oligodendrocytes, because the two proteins co-exist in both cell types in MSA [48, 58, 83, 94].

This supports the wider hypothesis that other brain proteins than p25α, e.g., tau, and metabolic and gut-derived factors [5, 30, 87, 88] sculpt the folding of α-Syn polymorphs, while the disease is progressively spreading through different cellular populations. This would suggest that a Braak stage 2 PD patient may be affected by strains induced by gut-associated factors, but can change strains in Braak stage 4 where the milieu of the dopaminergic neurons can affect α-Syn aggregates in the nigro-striatal pathway [64, 91], which might in turn differ from those affecting the neocortex in Braak stage 5–6.

A trivial non-structure-based factor that can confound seeding-based cell and in vivo experiments is whether the number of seeds differs significantly between preparations of strains [1]. The two preparations used in this study were sonicated prior to usage and validated both by determination of protein concentration and of size distribution of particles by DLS. We observed that the α-Syn/p25α strain consistently yielded larger particles than the α-Syn control strain. We ascribe this to either a larger flexibility or larger stability of the α-Syn/p25α aggregates, which renders them less prone to breakage when subjected to ultrasound. This should result in a lower number of particles when using equal amounts of filaments as starting material. Despite a potentially lower particle number, we consistently observed stronger effects of the α-Syn/p25 α polymorph across cell and animal models, which corroborates the existence of truly structure-dependent effects.

In cell models, the α-Syn/p25α strain templated larger inclusions when added to α-Syn-expressing cells ranging from mitotic oligodendroglial OLN-AS7 cells to human neuronal stem cell-derived neurons. The effect was likely caused by their intracellular templating activity, because both strains were equally well taken up by OLN cell. Rather, it reflects a different proteostatic handling of the intracellular aggregates being templated by the two polymorphs. This resembles the α-Syn-polymorph-specific inclusions seeded in iPSC-derived human cortical neurons [34] and in HEK293T cells expressing α-Syn fused to fluorescent reporter proteins [116].

To compare the functional effects of the α-Syn/p25α and the α-Syn control strains in vivo, we used both the hA53T-transgenic M83 line and wild-type C57BL/6 mice, and observed that the α-Syn/p25α strain induced differential α-Syn inclusion pathology, motor phenotypes, and time-to-death in the models. Inoculation of heterozygous M83 mice with the α-Syn/p25α strain decreased the disease-free period and induced a more aggressive disease phenotype as defined by a 45% shorter time from first symptom to death. This resembles the more aggressive disease observed when injecting PFF amplified from MSA brain [54]. The α-Syn/p25α strain induced more prominent pathology revealed by pS129 and p62 sequestrome-1 immunoreactivity reminiscent of MSA compared to PD [55]. The inclusions induced by the α-Syn/p25α strain displayed a differential binding of especially N-terminally directed α-Syn antibodies, which suggests a different structure of the aggregates formed by the two strains. The epitopes in the neuronal pathology induced by the two strains differed qualitatively in the brain stem and the red nucleus. However, quantitative analysis of the positivity for the DAB-based chromogen in the brain stem and the red nucleus for the N-terminal-directed 2H6 and pS129-directed 81a antibodies did not reveal a significant difference. This may suggest that the pathology induced by α-Syn PFF compared the α-Syn/p25α strain results in smaller aggregates that are more dispersed in the tissue and thus not appreciated by the qualitative inspection for more prominent cell body pathology.

To determine if we could find a biochemical or structural correlation of the aggregates or seeds in the tissue with the disease phenotype and inoculum of the strains, we conducted a series of experiments based on extracts of the pons and medulla oblongata of end-stage mice and age-matched healthy controls. First, we analysed the level of α-Syn aggregates based on an immune-FRET assay and demonstrated a significantly higher level of aggregates in the α-Syn/p25α strain induced mice that correlated with the stronger inclusion pathology. By contrast, the control α-Syn strain induced a low response close to the absent signal in the negative control animals. The higher aggregate load in the α-Syn/p25α strain-induced mice resembles the higher load in brain homogenates from rats expressing human α-Syn inoculated with MSA-derived strains compared to PD and DLB [106]. Second, using the PMCA technique, we amplified the seeds to demonstrate if the in vivo generated seeds retained structural and biochemical characteristics of the inoculated strains. Surprisingly, the analysis demonstrated that the seeding activity in the brain homogenates of the α-Syn/p25α strain-induced animals was lower than in α-Syn strain-induced mice as determined by diluting the brain homogenate. ThT analysis of the amplified aggregates demonstrated that the maximal signal of the plateau differed within the groups and did not allow us to discriminate between groups. Moreover, the proteinase K digestion pattern also differed within the groups and did not allow us to discriminate between groups. The ThT analysis was repeated on aggregates reamplified from the primary amplificates and demonstrated no difference between the two groups.

Hence, the aggressive disease phenotype correlated with the load of aggregates, but could not be associated with any of the structural characteristics we tested. This was not entirely surprising, because it has previously been demonstrated that transgenic M83 mice expressing human A53T-α-Syn generate only Campbell–Switzer-silver positive inclusions when inoculated with MSA brain extracts [55]. Since LB are only positive for Campbell–Switzer-silver staining, whereas CGI in human MSA tissue are positive for both Campbell–Switzer- and Gallyas-silver-staining, this suggests that human A53T-α-Syn expressed by the mPrP promoter affects the fidelity of the templating.

Compared to the control α-Syn polymorph, the α-Syn/p25α strain exhibited a differential targeting in the CNS where especially the red nucleus and the reticular formation were affected. This suggests that the CNS spreading governed by α-Syn/p25α seeds depends not just on the α-Syn expression level and neuronal connectivity [39], but is also modulated by strain-dependent factors, e.g., binding of seeds to specific surface receptors [13, 63, 93]. Systematic investigations of ligands for aggregated and even pS129-phosphorylated α-Syn have been conducted [8, 67], but α-Syn strain-specific interactomes have still not been reported.

Prior studies of injection of human α-syn PFF into the mouse striato-nigral system have resulted in small/moderate dopaminergic death in SN, with subtle-to-no motor defects [60, 76, 81]. However, murine α-syn PFF injection can lead to neuronal dysfunction without significant cell death, but still induce relevant behavioural changes [99]. In wild-type mice, unilateral injection of the two strains into the striatum induced a hyperactivity that was detectable on the challenging beam after 6 months. In accordance with this, we have recently observed hyperactivity upon injection of murine α-syn PFF into the striatum of rats [102]. Such hyperactivity has been previously described as an early sign of neurodegeneration in α-Syn-transgenic mice and it is linked to increased dopamine in the striatum [52, 104], likely caused by disturbances in dopamine transporters in the nerve terminals [33]. The cylinder test allowed us to discriminate the two strains as early as 3 months after injection, where the α-Syn/p25α strain induced a stronger asymmetry of the use of the forelimbs that became apparent also in the hindlimbs at 6 months. Remarkably, the enhanced motor impairment of the α-Syn/p25α strain was not associated with a wider α-Syn aggregate pathology in the brain, but with a more localized α-syn pathology in SN. The control α-Syn strain induced a faster and more widespread development of inclusion pathology that was evident at 3 months in all regions connected to the striatum. By contrast, the α-Syn/p25α strain primarily targeted the striatum (area of injection) and the SN but with a lower load of inclusions than the control strain at earlier time points. However, after 6 months, the pathology induced in striatum and SN by the control α-Syn strains was almost completely resolved as previously reported [39], whereas the α-Syn/p25α-induced inclusions persisted in SN. This may indicate that the neurons had more difficulty in resolving the α-Syn/p25α-induced aggregates and thus corroborates our observation of larger inclusions in the cell models. As previously shown, α-Syn injections induced neuroinflammatory responses as revealed by the observed astrogliosis and microgliosis, as well as the MHCII upregulation, a key protein in PD immune response [37, 38, 102]. However, further suggesting a distinctive strain, this immune response was lower in the α-Syn/p25α mice. Indeed, the different inflammatory capacity of α-Syn has been associated with its aggregation and with mutations [24], which could affect its interaction with receptors, subsequent phagocytosis, and MHCII presentation by the microglia [24, 42]. This could explain the lower gliosis and MHCII expression and the trend to show less phagocytic marker CD68 in the α-Syn/p25α group. However, the nigral loss of dopaminergic neurons after 6 months was comparable for the two strains. This suggests that the behavioural deficits induced by the α-Syn/p25α strain were caused by neuronal dysfunctions rather than loss of neurons or inflammation-induced changes. The morphology of the neuronal inclusions induced by the two strains in the wild-type mice differed with the control α-Syn strain inducing more skein-like inclusions often localised on one side of the nucleus like LB inclusions. By contrast, the α-Syn/p25α strain induced large spherical and ring-like inclusion in the SN pars compacta. Similar neuronal inclusions in the M83 line were recently reported using PFF amplified from brain tissue of MSA patients, spontaneously sick M83 mice and de novo generated PFF [54]. The morphology of the LB-like inclusion induced by our control PFF suggests that the neurons were able to sequester the templated α-Syn aggregates in one area of the cell body. By contrast, the inclusions templated by the α-Syn/p25α strain filled the entire cell body and suggests an inefficient cellular sequestering of the aggregates that potentially remain free to engage in more toxic interactions in the neuron. The α-Syn/p25α-induced inclusions are reminiscent of the neuronal cytoplasmic α-Syn inclusions in the limbic regions in atypical MSA patients [3] that correlate with cognitive impairment [69]. The disconnect between inclusion burden and dysfunctions in the wild-type mice inoculated with our two strains suggests the existence of templated aggregates that despite exerting toxic effects are not detectable with our current immunohistochemical techniques that preferentially detect inclusions. This is corroborated by the demonstration of abundant α-Syn aggregate pathology in MSA detectable by the proximity ligation assay, which did not overlap with GCI detectable by immunohistochemistry [82, 90].

Our results demonstrate that the oligodendroglial protein p25α can stimulate folding of an α-Syn aggregate strain that in vivo templates neuronal α-Syn aggregates with enhanced neurodegenerative potential as evidenced by earlier motoric dysfunctions and a more aggressive disease course. The interaction between p25α and α-Syn may represent a novel target in MSA and the in vitro generated α-Syn/p25α polymorph, a novel tool for modelling synucleinopathies. There is no reason to expect this example of strain-specific folding dictated by a particular binding partner to be an isolated case. p25α was originally identified in rat brain cytosolic extract through its ability to associate with α-Syn aggregates [58], and co-aggregates of α-Syn and tau can also seed α-Syn aggregation in vivo albeit with less spreading of pathology compared to α-Syn aggregates alone [113]. Insight into the cellular location and impact on aggregation of these proteins may open new perspectives in our understanding of strain development and how to combat this devastating class of diseases.

## Supplementary Information

Below is the link to the electronic supplementary material.

Supplementary file1 (PDF 49095 kb)

## Data Availability

The data that support the findings of this study are available from the corresponding authors upon reasonable request.
