# Supplementary material for: Multiple system atrophy-associated oligodendroglial protein p25α stimulates formation of novel α-synuclein strain with enhanced neurodegenerative potential
Source: Acta Neuropathol. 2021 May 12;142(1):87–115. doi: 10.1007/s00401-021-02316-0 (PMC8217051; doi:10.1007/s00401-021-02316-0)
Supplement: Supplementary file 1 — Supplementary file1 (PDF 49095 kb) [file 401_2021_2316_MOESM1_ESM.pdf]

Electronic Supplementary Material for:

Multiple system atrophy-associated oligodendroglial protein p25α stimulates formation of novel α-synuclein strain with enhanced neurodegenerative potential.

Nelson Ferreira[1*], Hjalte Gram[1], Zachary A. Sorrentino[2], Emil Gregersen[1], Sissel Ida Schmidt[3], Lasse Reimer[1], Cristine Betzer[1], Clara Perez-Gozalbo[1], Marjo Beltoja[1], Madhu Nagaraj[4], Jie Wang[4,5], Jan S. Nowak[4], Mingdong Dong[4], Katarina Willén[6], Ersoy Cholak[6], Kaare Bjerregaard-Andersen[6], Nicolas Mendez[7], Prakruti Rabadia[7], Mohammad Shahnawaz[7], Claudio Soto[7], Daniel E. Otzen[4], Ümit Akbey[4,8,9], Morten Meyer[3,10], Benoit I. Giasson[2], Marina Romero-Ramos[1], Poul Henning Jensen[1*]

[1] DANDRITE, Danish Research Institute of Translational Neuroscience & Department of Biomedicine, Aarhus University, 8000 Aarhus C, Denmark.

[2] Department of Neuroscience, Center for Translational Research in Neurodegenerative Diseases and McKnight Brain Institute, University of Florida, Gainesville, USA.

[3] Department of Neurobiology Research, Institute of Molecular Medicine, University of Southern Denmark, J.B. Winsloews Vej 21, st, DK5000 Odense C, Denmark.

[4] Interdisciplinary Nanoscience Center (iNANO), Aarhus University, DK-8000 Aarhus C, Denmark.

[5] Institute for Advanced Materials, School of Material Science and Engineering, Jiangsu University, Zhenjiang 212013, China.

[6] Department of Cell Biology, H. Lundbeck A/S, Valby, Denmark.

[7] Mitchell Center for Alzheimer's Disease and Related Brain Disorders, Department of Neurology, University of Texas McGovern Medical School at Houston, Houston, TX, USA.

[8] Aarhus Institute of Advanced Studies (AIAS), Aarhus University, 8000 Aarhus C, Denmark

[9] Institute of Complex Systems (ICS6), Structural Biochemistry, Research Center Julich, 52415, Julich, Germany.

[10] BRIDGE, Brain Research – Inter-Disciplinary Guided Excellence, Department of Clinical Research, University of Southern Denmark, J.B. Winsloews Vej 19, DK5000 Odense C, Denmark.

Supplementary Fig. 1

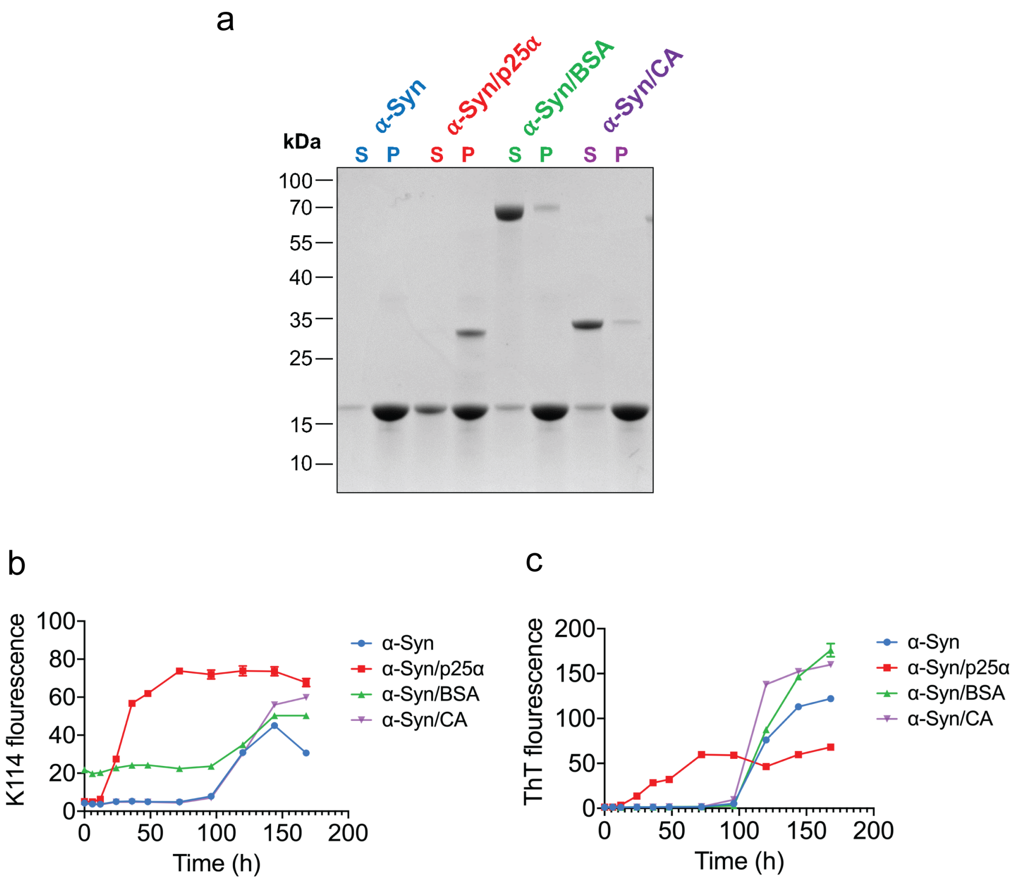

Supplementary Fig. 1 - Fibril assembly and sedimentation of α-Syn incubated with p25α, bovine serum albumin or carbonic anhydrase. Soluble monomeric wild-type α-Syn (277µM) was purified through a 100kDa filter (Amicon® Ultra 100K device, Merck) and incubated with recombinant p25α (13.85 µM), bovine serum albumin (BSA, 13.85 µM) or carbonic anhydrase (CA, 13.85 µM) at 37°C in phosphate-buffered saline pH 7.4 (PBS, Gibco) with continuous shaking at 1050 r.p.m. (Eppendorf Thermotop) for up to 168 h. **a)** At end-stage, supernatant (S) and pellet (P) fractions were analysed on a Coomassie Blue stained SDS-polyacrylamide gel to evaluate solubility by centrifugation. During the incubation, samples were removed and analysed for amyloid formation by ThT and K114 fluorescence. **b)** K114 and **c)** ThT fluorescent amyloid assay. Y-axis demonstrates the ThT or K114 fluorescence in arbitrary units. Data represents mean ± s.d. of duplicate samples in one representative experiment from three independent experiments.

Supplementary Fig. 2

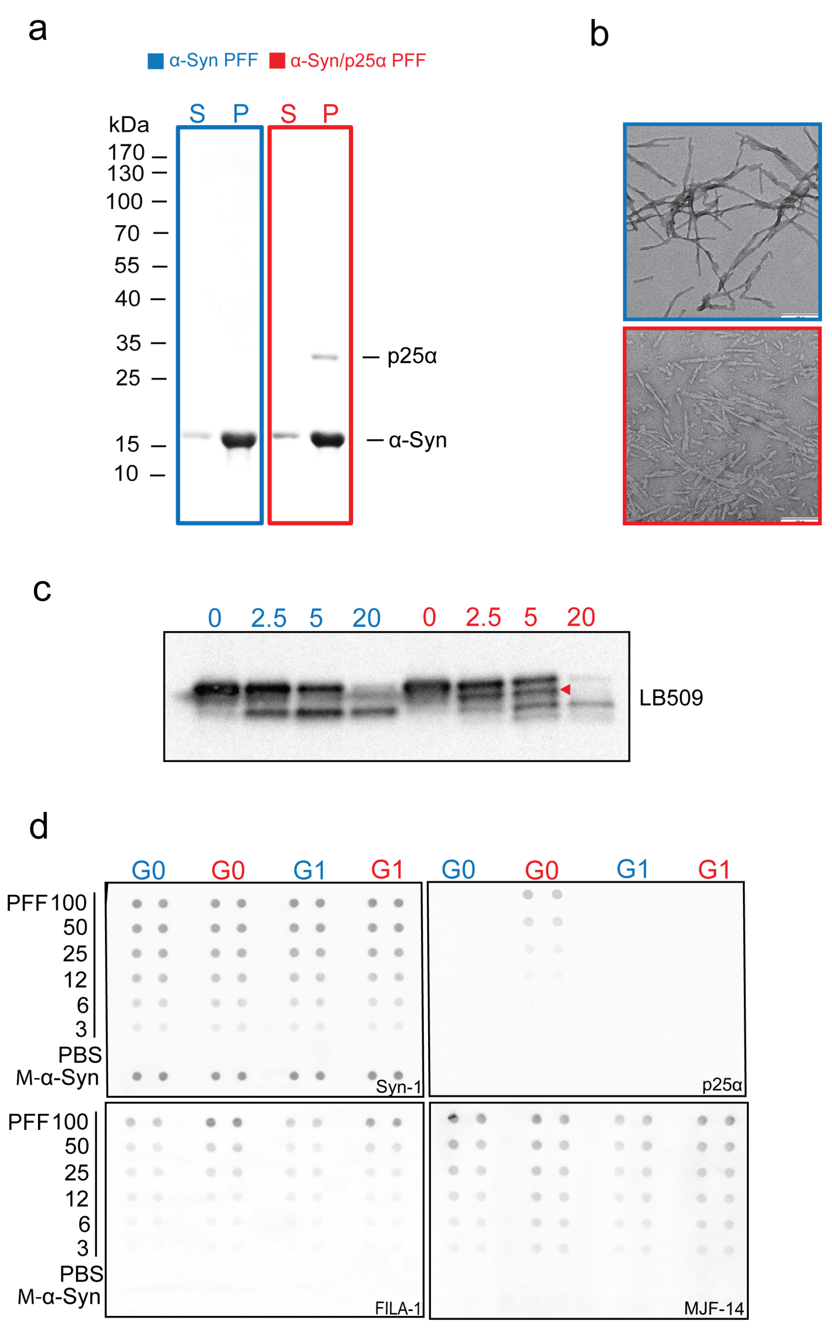

Supplementary Fig. 2 - Biochemical and structural comparison of α-Syn and α-Syn/p25α strains. Soluble monomeric α-Syn (346 μM) was assembled in the absence or presence of p25α (17 μM) into PFF by incubation at 37°C in phosphate-buffered saline pH 7.4 (PBS, pH 7.4, Gibco) with continuous shaking at 1050 r.p.m. (Eppendorf Thermotop). The generated PFF were harvested by centrifugation 15,600 *g* at 25°C for 30 min, and supernatants (S) and pellets (P) were subjected to: **a)** Denaturation followed by SDS-PAGE and Coomassie Blue staining to demonstrate the purity of the preparation and presence of 5% p25α in the *de novo* generated α-Syn/p25α PFF. Isolated PFF were analysed by **b)** transmission electron microscopy (TEM. Scale bar = 200 nm. **c)** Proteolytic digestion with increasing concentrations of proteinase K (up to 20 μg/ml) were resolved by SDS-PAGE and subjected to immunoblotting with LB 509 antibody (Abcam, aa 115-122). Red arrow head indicates α-Syn/p25α PFF-specific proteinase K-generated band. **d)** Native dot blotting of *de novo* formed (G0) and reamplified (G1) PFF with aggregate-specific MJFR-14 and FILA-1 antibodies, pan-α-Syn Syn-1 antibody (BD Biosciences) and rabbit anti-p25α antibody. PFF was immobilised in PBS (pH 7.4, Gibco) solvent control. Syn-1 demonstrates equal loading, and anti-p25α demonstrates that p25α only is detectable in the G0 but not reamplified G1 α-Syn/p25α PFF.

Supplementary Fig. 3

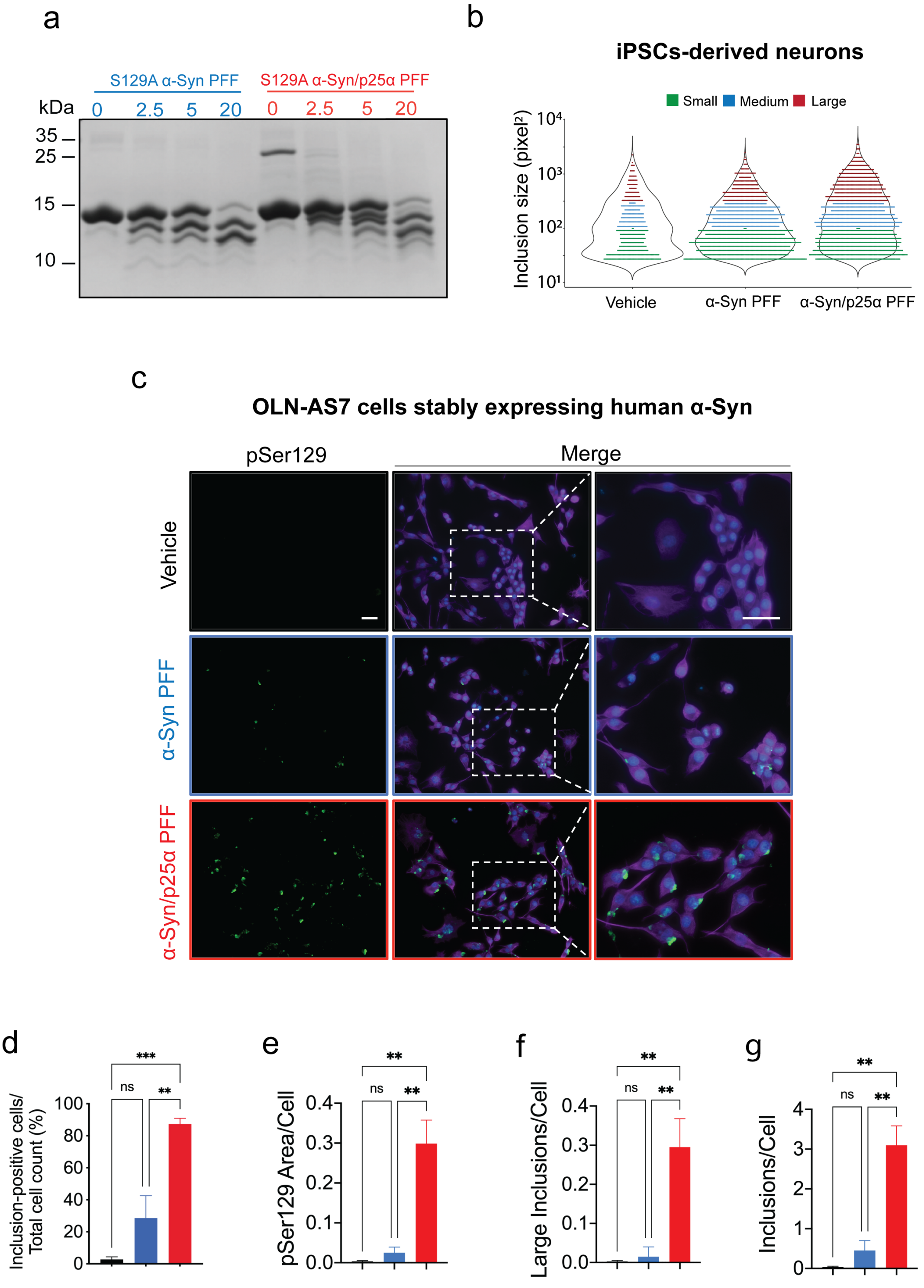

Supplementary Fig. 3 - α-Syn/p25α PFF enhances templated inclusion formation in two different cell models. **a)** *De novo* generated S129A α-Syn (labelled in blue) and α-Syn/p25α (labelled in red) were isolated by centrifugation and incubated with increasing concentrations of proteinase K, indicated in μg/ml above the gels and resolved by SDS-PAGE followed by Coomassie Blue staining. Molecular size standards in kDa are indicated to the left of the gel. **b)** Violin plot showing inclusion size and inclusion diameter distribution in human iPSCs-derived neurons displayed in panel Fig. 3d. **c-g)** Rat oligodendroglial cells (OLN-AS7) stably expressing human α-Syn were incubated with 14 μg/ml of α-Syn or α-Syn/p25α PFF in cell medium for 12 h followed by a washing step to remove PFF excess. Cells subsequently grew for 36 h before being subjected to immunocytochemistry. **c)** **L**eft panels: pSer129; middle panels: pSer129 and DAPI-stained nuclei; right panels: enlarged boxes from middle panel. Scale bar = 50 μm. The scale bar in top left panel applies left and middle columns, and in top right panel to the right column. Bar graphs illustrate normalised **d)** Inclusion-positive cells/Total cell count, **e)** p-S129 Area/cell, **f)** Large inclusions (>142.8 μm^2)/cell and **g)** Inclusions/cell. Data in panels d-g are shown as mean of three independent experiments ± s.e.m. ns: not significant. $^{**}P < 0.01$. One-way ANOVA followed by Tukey's multiple comparisons test.

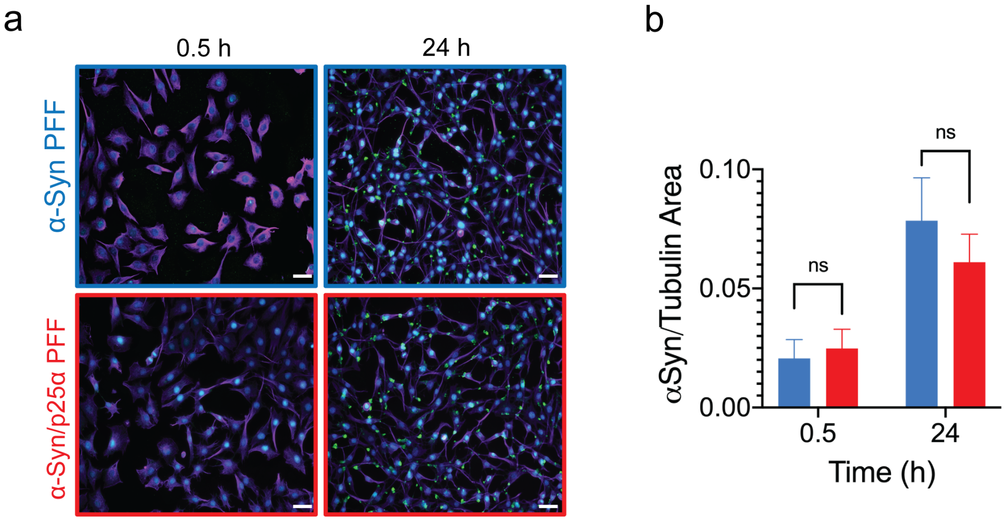

Supplementary Fig. 4 - α-Syn and α-Syn/p25α PFF are internalised equally well in oligodendroglial cells. Rat oligodendroglial cell line OLN-93 not expressing human α-Syn, maintained as aforementioned in Supplementary Fig. 3c-g, were treated for 0.5 or 24 h with 14 µg/mL of S129A-α-Syn PFF or S129A-*α*-Syn/p25α at 37 ºC. The cells were subsequently washed three times with HBSS to remove unincorporated PFF and then fixed in 4% PFA. **a)** To visualize all internalized PFF, the coverslips were subjected to immunostaining with primary antibodies against total α-Syn (ASY-1, 1:1000,) and tubulin (1:1000, Abcam #ab6160) and corresponding secondary Alexa Flour antibodies and 4′,6-diamidino-2-phenylindole (DAPI) (1:10,000, Th.Geyer). Images were acquired with a Zeiss Observer.Z1 microscope, and image analysis was performed using ImageJ. Scale bar = 50 µm **b)** Bar graphs illustrate normalised α-Syn/Tubulin area. Data represents *n*=3 independent experiments ± s.e.m. ns: not significant. One-way ANOVA followed by Sidak's multiple comparisons test.

■ Vehicle ■ α-Syn PFF ■ α-Syn/p25α PFF

a

pSer129

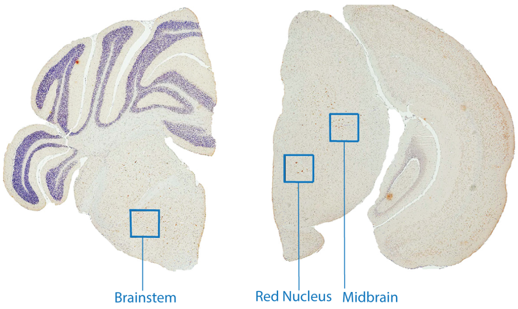

b

Area	Vehicle	α-Syn/p25α PFF	αSyn PFF
Reticular formation	0/7	7/7	7/7
Cerebellum	0/7	3/7	4/7
Periaqueductal gray	0/7	6/7	7/7
Deep mesencephalic nucleus	0/7	7/7	7/7
Red nucleus	0/7	6/7	5/7
Superior colliculus	0/7	5/7	4/7
Ventral thalamus	0/7	7/7	6/7
Hypothalamus	0/7	3/7	2/7
Amygdala	0/7	2/7	2/7
Motor cortex	0/7	0/7	1/7

c

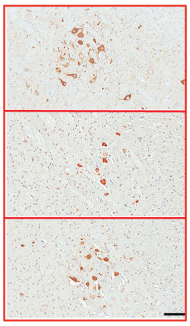

e

Antibody	Epitope	α-Syn/p25α PFF Summary	α-Syn PFF Summary	Cohort difference
LS4-2G12	α-Syn pSer129	Widespread pathology	Widespread pathology	More inclusions in RN for α-Syn/p25α
81A	α-Syn pSer129	Widespread pathology	Widespread pathology	More inclusions in RN for α-Syn/p25α
EP1536Y	α-Syn pSer129	Widespread pathology	Widespread pathology	More inclusions in RN for α-Syn/p25α
P62-Sequestosome	Sequestosome	Widespread pathology; less staining of processes	Widespread pathology; less staining of processes	More inclusions in RN for α-Syn/p25α
Ubiquitin 1	Ubiquitin	Widespread pathology	Widespread pathology	More inclusions in RN for α-Syn/p25α
AT8-7F2	pThr205 tau	No staining	No staining	None
Syn506	Conformation selective 1-12 α-Syn	Widespread pathology	Widespread pathology	More inclusions in RN for α-Syn/p25α PFF
GFAP	Astrocytes	Widespread gliosis paralleling pSer129	Widespread gliosis paralleling pSer129	None
Cd11b	Microglia	Widespread gliosis paralleling pSer129	Widespread gliosis paralleling pSer129	More gliosis in RN for α-Syn/p25α
94-3A10	130-140 α-Syn	More restricted pathology; RF, midbrain, RN	More restricted pathology; RF, midbrain, RN	More inclusions in RN for α-Syn/p25α
15-4E7	130-140 α-Syn	Widespread pathology	Widespread pathology	More inclusions in RN for α-Syn/p25α
94-3B2	102-110 α-Syn	Rare staining only in RN	No staining	More inclusions in RN for α-Syn/p25α
1F11	2-21 α-Syn	Rare staining only in RF	Rare staining only in RF	None
1D12	2-21 α-Syn	More restricted pathology; RF, midbrain, RN	More restricted pathology; RF, midbrain, RN	More inclusions in all areas for α-Syn/p25α
9C10	2-21 α-Syn	More restricted pathology; RF, midbrain, RN	More restricted pathology; RF, midbrain, RN	More inclusions in RN for α-Syn/p25α
2H6	2-21 α-Syn	More restricted pathology; RF, midbrain, RN	More restricted pathology; RF, midbrain, RN	More inclusions in all areas for α-Syn/p25α

d

GFAP

Brainstem Midbrain Red Nucleus

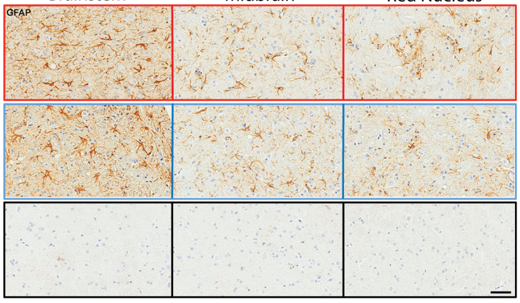

f

α-Syn 2-21 (1D12)

Brainstem Midbrain Red Nucleus

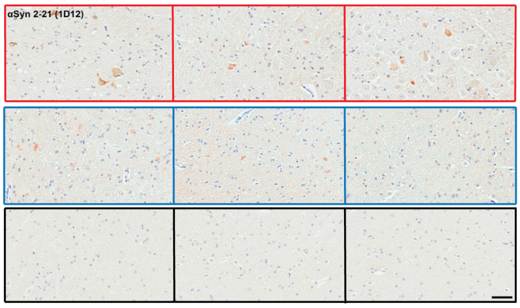

g

Area	Vehicle	α-Syn/p25α PFF	αSyn PFF
Reticular formation	0/7	7/7	7/7
Cerebellum	0/7	0/7	0/7
Periaqueductal gray	0/7	0/7	0/7
Deep mesencephalic nucleus	0/7	5/7	4/7
Red nucleus	0/7	5/7	1/7
Superior colliculus	0/7	0/7	0/7
Ventral thalamus	0/7	0/7	0/7
Hypothalamus	0/7	0/7	0/7
Amygdala	0/7	0/7	0/7
Motor cortex	0/7	0/7	0/7

Supplementary Fig. 5 - Immunohistochemical evaluation of CNS α-Syn and associated pathology in end-stage M83 mice injected with α-Syn or α-Syn/p25α PFF. a) Low-magnification-representative coronal sections of α-Syn PFF-injected mice stained with the antibody against pSer129 LS4-2G12. Boxes indicate locations of magnified regions in Fig. 4e. **b)** Semi-quantitative pathology scoring summary using pSer129 α-Syn between the three cohorts in this study; the number of mice with detectable pSer 129 α-Syn pathology in a given region (n/7) is shown along with density of pathology in that region represented by colour where darker colour indicates more pSer129-αSyn-reactive inclusions. **c)** The red nucleus for three separate mice injected with α-Syn/p25α PFF; most mice in the α-Syn-p25α cohort had abundant red nuclear pathology with unique inclusion morphology not detected in the other two cohorts. Scale bar = 100 μm. **d)** Representative images of immunohistochemical analysis depicting astrocytosis in the brainstem, midbrain and red nucleus using an antibody to GFAP. Scale bar = 50 μm. **e)** A table depicting the antibodies used in this study and the summary of pathology findings from staining 3-7 mice of each cohort along with any differences between the cohorts when stained with each antibody; PBS-injected mice are not shown as no inclusions or inflammation were detected in any of those mice. **f)** Representative images of immunohistochemical analysis of N-terminal reactive α-Syn in the brainstem, midbrain and red nucleus using the 1D12 monoclonal antibody against αSyn 2-21. Scale bar = 50 μm. **g)** Semi-quantitative pathology scoring summary using 2H6 antibody staining between the three cohorts in this study; the number of mice with detectable 2H6-positive pathology in a given region (n/7) is shown along with density of pathology in that region represented by colour where darker colour indicates more 2H6-reactive inclusions. Two monoclonal N-terminal antibodies in this study, 2H6 and 1D12, display more restricted staining of inclusions than pSer129, and more inclusions are detected using these antibodies in the α-Syn/p25α cohort than with α-Syn fibrils alone.

Supplementary Fig. 6

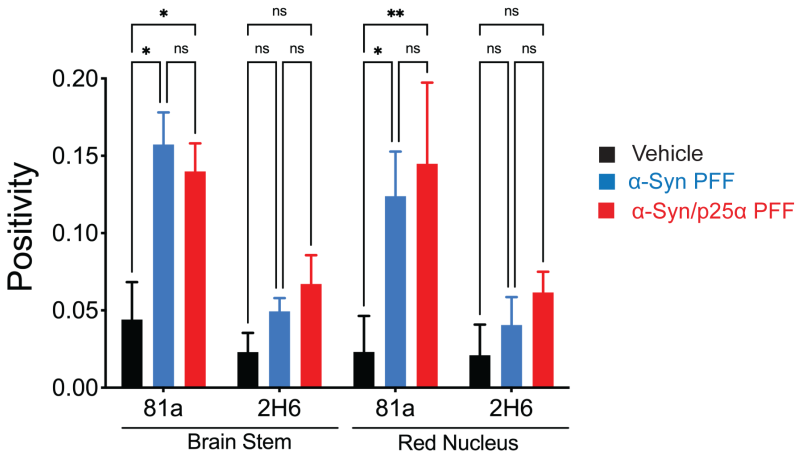

Supplementary Fig. 6 - Quantitative analysis of α-Syn and α-Syn/p25α PFF induce staining for 81a and 2H6 positivity in brain stem and red nucleus. Graph depicting the positivity (proportion of pixels with positive staining) for the brain stem (Br) and red nucleus (RN) for each of the three cohorts in this study (PBS, α-Syn and α-Syn/p25α). Positivity is shown for both 81a and 2H6 antibody staining. Error bars 95% CI. Two-way ANOVA followed by Tukey's posthoc. ns: not significant. **P* < 0.05, ***P* < 0.01.

Supplementary Fig. 7

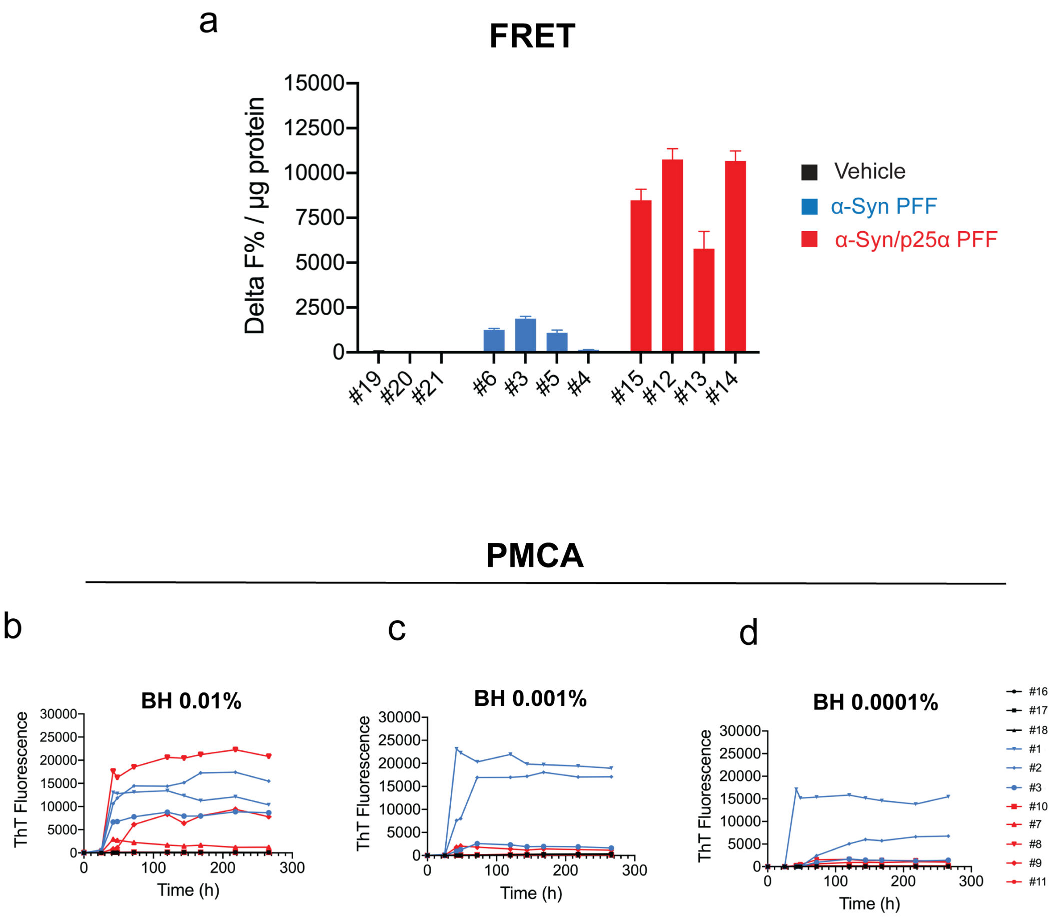

Supplementary Fig. 7 - Detection of α-Syn aggregates and templating active seeds in individual mouse brain samples.

a) Aggregated α-Syn was quantified in brain homogenates using a FRET based Cisbio assay (PerkinElmer) with three technical replicates per homogenate. The level of aggregated α-Syn in the samples is expressed as Delta F% /ug protein (vehicle PBS, *n*=3, mice #19-21; α-Syn PFF, *n*=4, mice #3-6; α-Syn/p25α PFF *n*=4, mice #12-15). BCA protein measurements determined the total protein concentration of the homogenates. Neuropathological information of mice samples is available in Supplementary Table 2. **b-d)** Brain homogenates samples from mouse inoculated with α-Syn PFF (blue line, *n*=3, mice #1-3), α-Syn/p25 PFF (red line, *n*=5, mice #7-11) and vehicle (PBS, black line, mice #16-18) were homogenized at 10% w/v. Brain samples (at final concentrations of 0.01, 0.001 and 0.0001%) were added to the wells of a 96-well plate. α-Syn-PMCA assay was started by adding α-Syn monomers (1 mg/ml) and thioflavin-T (5 μM) in 100 mM PIPES pH 6.5 containing 500 mM NaCl. The plate was incubated at 37 °C with intermittent shaking at 500 r.p.m. The extent of aggregation was monitored by an increase in ThT fluorescence by a spectrofluorometer using an λ_{ex}=435 nm and an λ_{em}=485 nm. Neuropathological information of mice samples is available in Supplementary Table 2.

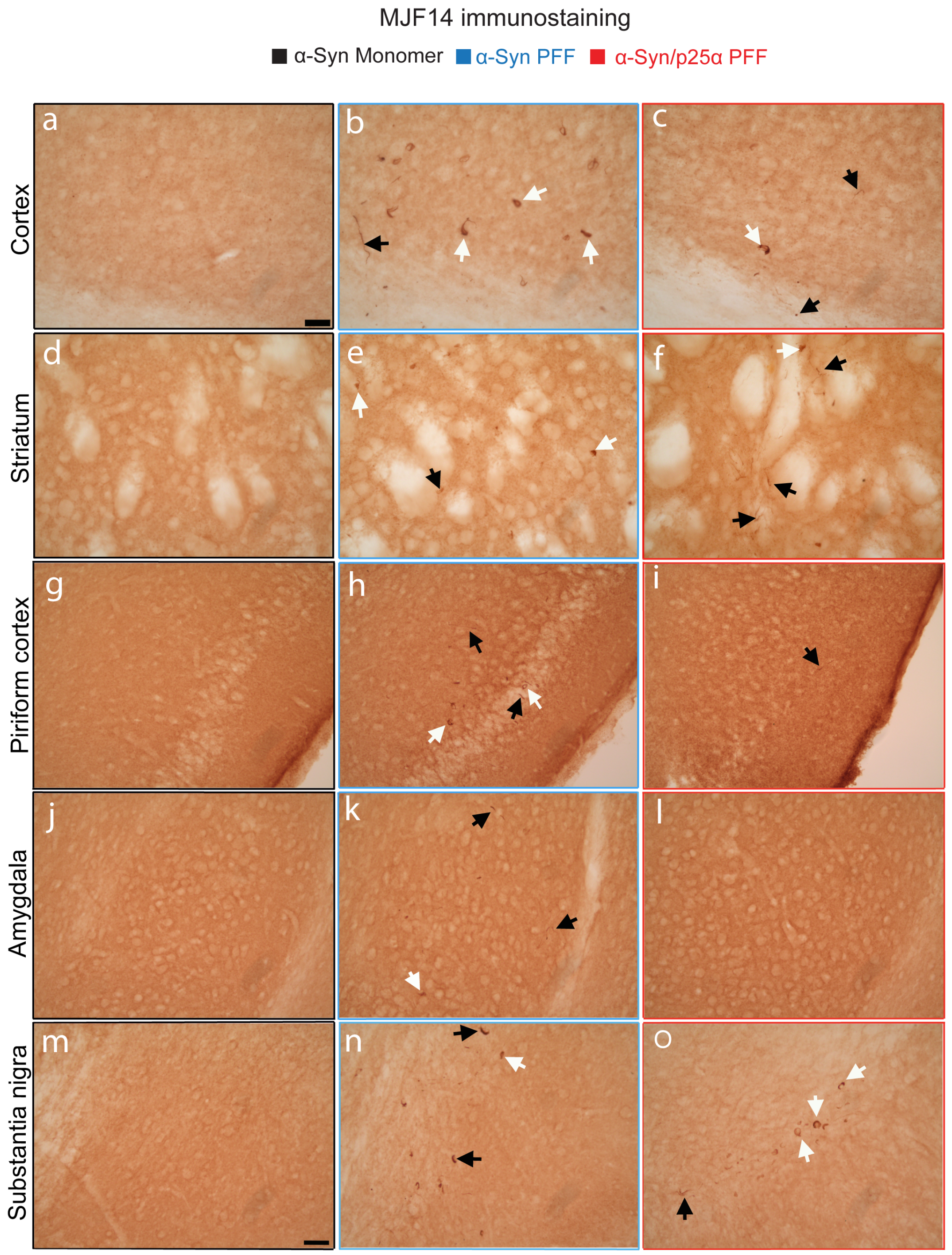
MJF14 immunostaining
α-Syn Monomer
α-Syn PFF
α-Syn/p25α PFF
Cortex
Striatum
Piriform cortex
Amygdala
Substantia nigra
a
b
c
d
e
f
g
h
i
j
k
l
m
n
o

Supplementary Fig. 8 - α-Syn pathology in mice injected into striatum with monomeric, α-Syn PFF or α-Syn/p25α PFF 3 months after surgery. Representative photos from brain sections immunostained with an antibody against aggregated α-Syn (MJF-14). While no obvious immunostaining was found in monomerically injected animals (black lined boxes), α-Syn PFF-injected mice (blue lined boxes) showed pathological α-Syn aggregation in the injection area, striatum and anatomically connected areas: cortex, piriform cortex, amygdala and SN. These structures appeared polygonal or with certain circularity (with arrows), such as half-moon shape or circular at times resembling a cell body. However, we also observed long fibre-like structures (black arrows). The mice injected with α-Syn/p25α (red lined boxes) showed much fewer pathological α-Syn structures in striatum and only few in cortex or piriform cortex. No MJF-14-positive staining was seen in this group in the amygdala. However, numerous cellular structures immunostained for MJF-14 were found in the substantia nigra pars compacta. Scale bar in a) is 25 µm and applies to cortex and striatum images. Scale bar in m) is 50 µm and applies to piriform cortex, amygdala and substantia nigra images.

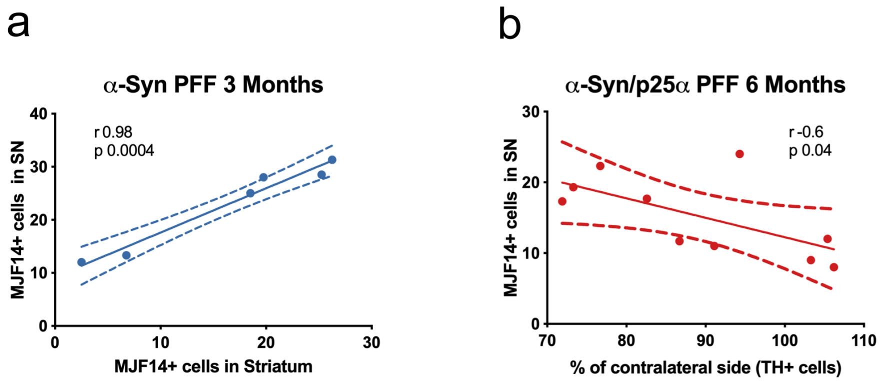

Supplementary Fig. 9 - Correlation analyses of mice inoculated with α-Syn PFF and α-Syn/p25α PFF. a) Number of MJF-14^{+} cells in SN and striatum at 3 months correlated in the α-Syn PFF-injected group (Pearson r). **b)** At 6 months, the number of MJF-14^{+} cells in SN in the α-Syn/p25α PFF was inversely correlated with the percentage of surviving dopaminergic cells as determined by comparison with the contralateral side (Pearson r).

Supplementary Fig. 10

a

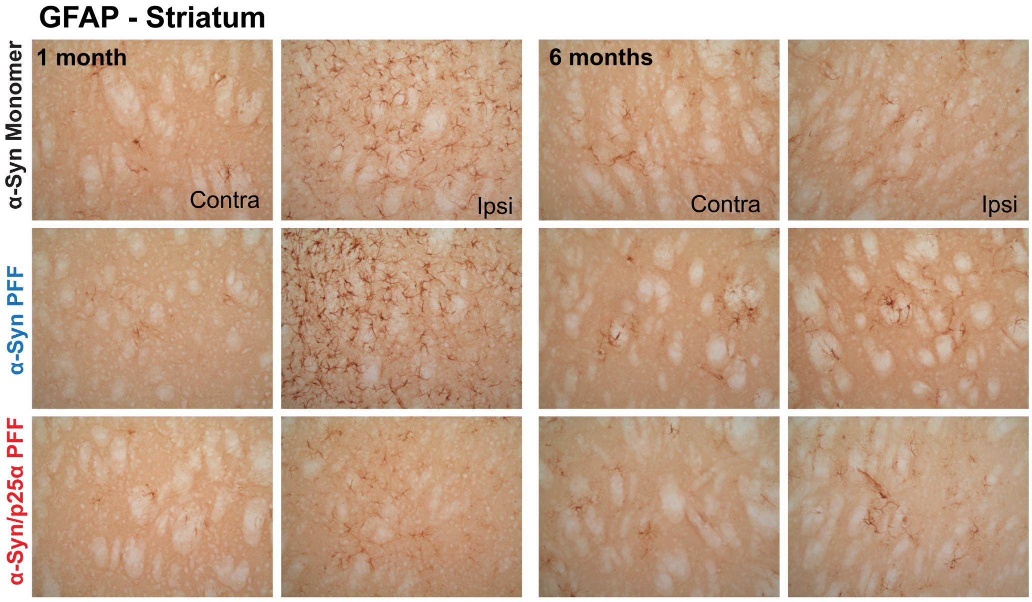

b

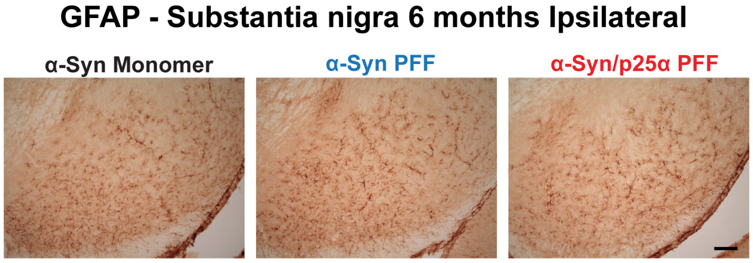

Supplementary Fig. 10 - GFAP expression in mice injected into striatum with monomeric, α-Syn PFF or α-Syn/p25α PFF at 1- and 6-months after surgery. a) Representative photos from brain sections immunostained with an antibody against GAFP. Both monomeric and α-Syn PFF showed significant increase of GFAP expression in the ipsilateral striatum at 1 month, with the α-Syn PFF animals showing the strongest immunostaining; the α-Syn/p25α PFF cohort showed the lowest number of cells and staining intensity at that time point. Few scattered cells expressing low/moderate levels of GFAP were seen in the contralateral striatum at 1 month in all groups. **b)** At 6 months, no major differences were seen across cohorts in the ipsilateral striatum that showed fewer cells than at 1 month. The contralateral striatum showed more cells after 6 months than after 1 month in all three groups. No major differences in the GFAP immunostaining were seen between groups in the ipsilateral substantia nigra after 6 months. Scale: 100µm for all substantia nigra images; 50 µm for all striatum panels.

Supplementary Fig. 11

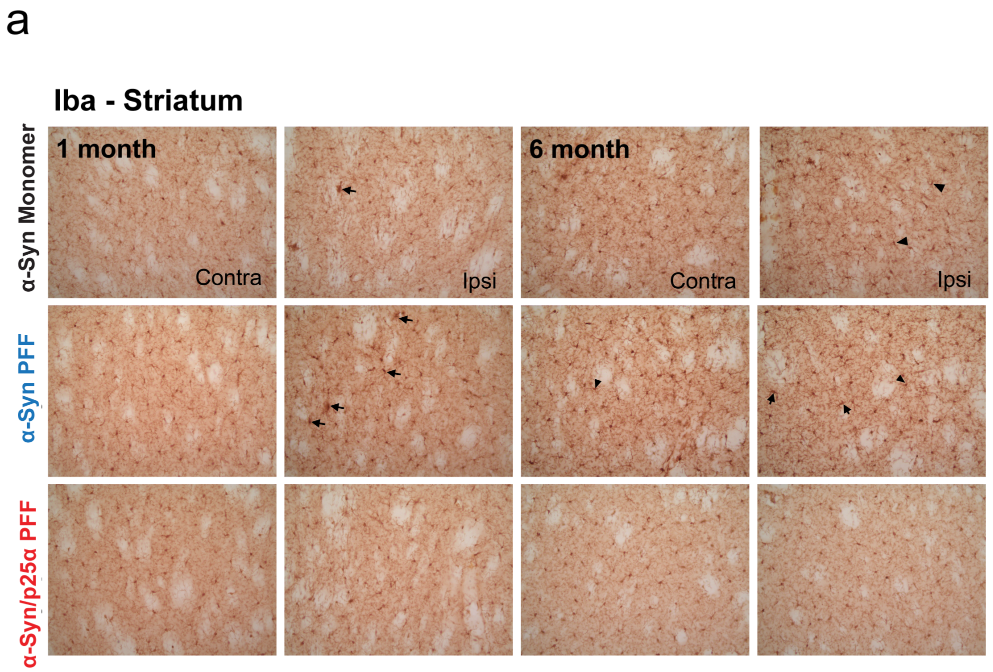

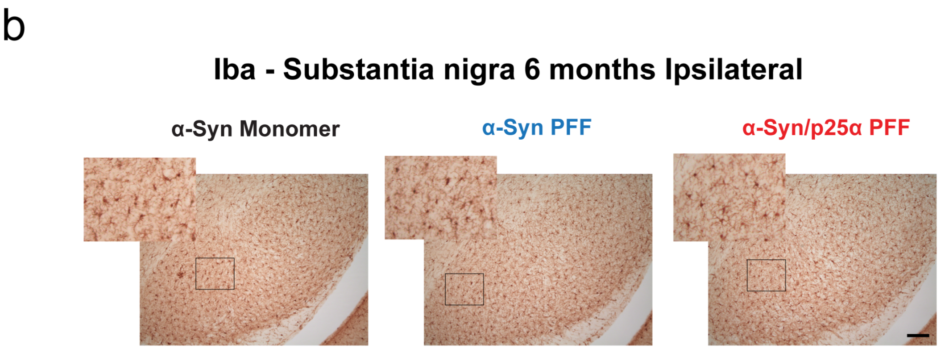

Supplementary Fig. 11 - Iba1 expression in mice injected into striatum with monomeric, α-Syn PFF or α-Syn/p25α PFF at 1 and 6 months after surgery. a) Representative photos from brain sections immunostained with an Iba1 antibody. At 1 month, all three groups showed more Iba1+ cells in the ipsilateral side than in the contralateral striatum - with some hypertrophic Iba1+ cells and cells of bigger cell body (arrows). However, this was more obvious in the monomeric and α-Syn PFF mice, with the α-Syn PFF animals showing the strongest immunostaining, while the α-Syn/p25α mice showed the lowest number of cells and staining intensity in the ipsilateral side among the three cohorts. **b)** At 6 months, we noticed an upregulation of the protein expression in the contralateral side of the striatum in monomeric and α-Syn PFF-injected mice, with the ipsilateral showing less hypertrophic (arrows) cells than at 1 month, but numerous hyper-ramified, strongly stained Iba1+ cells (arrow heads). The α-Syn/p25α PFF-injected mice showed no difference between ipsi- and contralateral sides of the striatum, with lower Iba1+ intensity than in other two groups. No apparent differences in the Iba1 immunostaining were seen between cohorts in the ipsilateral substantia nigra after 6 months. Insets show the areas framed in the substantia nigra images. Scale: 100µm for all substantia nigra images; 50 µm for all striatum panels.

Supplementary Fig. 12

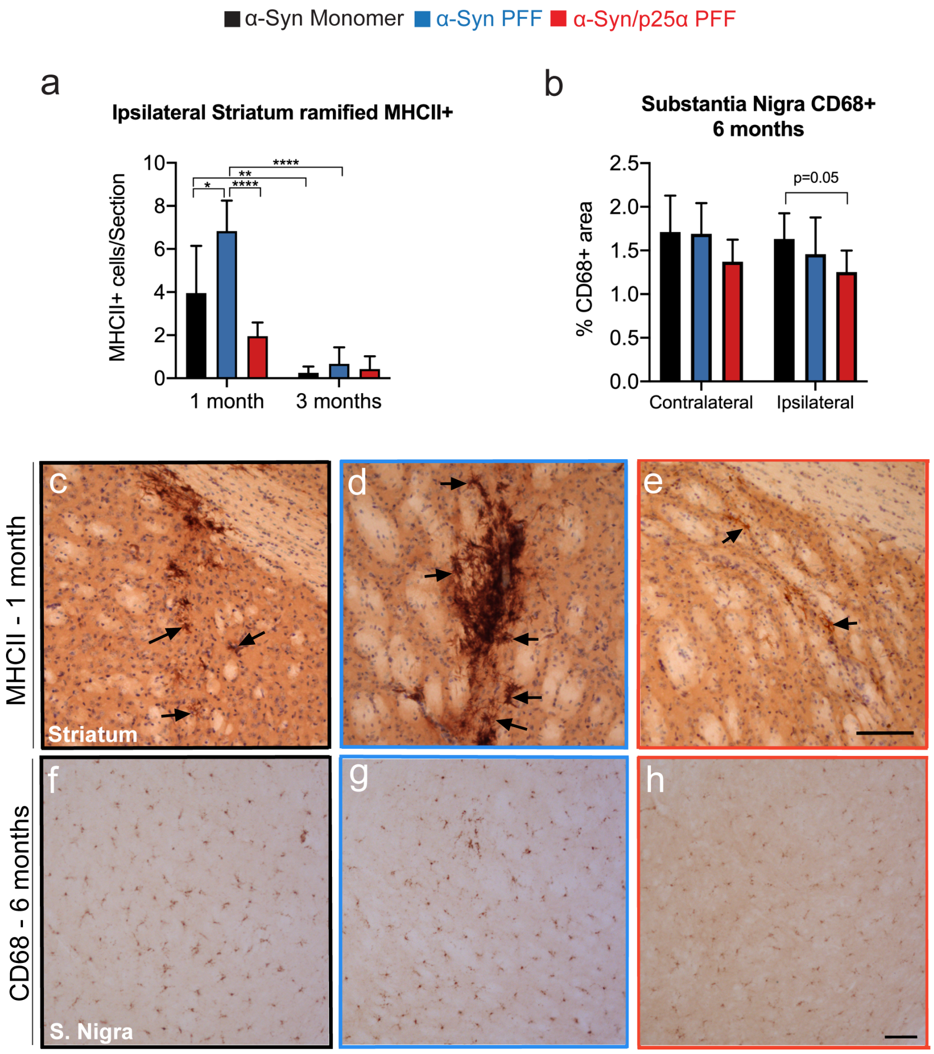

Supplementary Fig. 12 - Progression of brain immune response in mice injected with monomeric, α-Syn PFF or α-Syn/p25α PFF.

a) Brain sections were immunostained with an antibody against MHCII; ramified $MHCII^+$ cells were counted in several sections from the ipsilateral striatum; and the average number of cells per coronal section in each group was calculated at 1 and 3 months after surgery. Both monomeric (black-lined boxes) and α-Syn PFF (blue-lined boxes) showed significant increase of MHCII expression, with the α-Syn PFF animals showing a significantly higher number of $MHCII^+$ cells in striatum at 1 month; while the α-Syn/p25α (red lined boxes) animals showed the lowest number of cells at that time point. Both monomeric and α-Syn PFF showed a significant decrease of the $MHCII^+$ population after 3 months. **b)** Nigral sections (contra- and ipsilateral) from animals 6 months after surgery were CD68-immunostained and photographed, and the area covered with immunostaining was measured. Again, both monomeric and α-Syn PFF showed obvious CD68 expression, while the α-Syn/p25α PFF animals showed a trend towards having a lower CD68 expression in substantia nigra (vs. monomeric). **c-e)** Representative photos from striatal MHCII-immunostained sections at 1 month showing $MHCII^+$ cells (arrows) in the proximity of the injection area, and **(f-h)** photos of SN CD68-immunostained sections at 6 months. Notice the lower early and late immune activation on the α-Syn/p25α PFF mice (**e & h**) than in the other two groups (**c, d, f & g**). Two-way ANOVA followed by Sidak's posthoc. *$P < 0.05$, **$P < 0.01$, ***$P < 0.001$. Error bars indicate mean ± s.e.m. Scales: 50 μm in **e** applies to **c-e**, in **h** applies to **f-h**.

Supplementary Table 1

	Tuttle et al. 2016 bmrb #25518		Gath et al. 2012 bmrb #18860		Gath et al. 2014 bmrb #17498		Verasdonck et al. 2016 bmrb #25535		
Residue	Ca	Cb	Ca	Cb	Ca	Cb	Ca	Cb	Residue
9S	-	-	-	-	57.75	66.65			9S
22T	-	-	-	-	65.74	68.93			22T
33T	-	-	-	-	60.03	69.92			33T
42S	-	-	55.32	65.94	54.55	64.52	58.93	68.88	42S
44T	59.57	71.50	-	-	60.77	72.17	58.86	71.09	44T
54T	61.65	62.25	62.97	69.91	-	-	58.53	71.45	54T
59T	-	-	57.12	68.77	59.77	71.94	61.07	69.37	59T
64T	62.25	69.71	61.98	70.12	62.43	69.94	62.44	69.72	64T
72T	59.40	69.40	60.53	70.53	60.68	68.61			72T
75T	61.83	70.38	62.82	69.34	58.49	71.92	60.45	72.18	75T
81T	60.94	72.21	59.74	73.73	68.71	67.78	62.13	70.15	81T
87S	58.81	64.70	56.03	67.77	56.32	67.20	56.43	65.45	87S
92T	61.03	69.79	61.15	71.79	61.30	70.89	60.50	70.85	92T
	High-resolution ssNMR structure		"Fibril" ssNMR assg.		"Ribbon" ssNMR assg.		High-pH ssNMR assg.		

Supplementary Table 1. Chemical shift assignments for previously reported α-Syn aggregates.

Supplementary Table 2

Cohort	Mouse #	Onset of Disease (dpi)	Disease Duration (days)	Time of Death (dpi)
α-Syn PFF	1	120	42	162
	2	120	42	162
	3	104	41	145
	4	112	33	145
	5	112	42	154
	6	112	33	145
α-Syn/p25α PFF	7	72	24	96
	8	88	16	104
	9	80	24	104
	10	88	32	120
	11	96	24	120
	12	88	24	112
	13	88	24	112
	14	72	24	96
	15	80	16	96
Vehicle	16	-	-	180
	17	-	-	180
	18	-	-	180
	19	-	-	180
	20	-	-	180
	21	-	-	180

Supplementary Table 2. Behavioural information of TgM83$^{+/-}$ mice used for the biochemical FRET and PMCA analysis demonstrated in Supplementary Fig. 7. The mice were bilaterally injected into the gastrocnemius muscle with vehicle (PBS, pH 7.4, sacrificed at 180 d.p.i), α-Syn PFF or α-Syn/p25α PFF (sacrificed at terminal stage). Onset of disease was defined as onset of clasping. Disease duration was defined as time from the onset of clasping until time of death.
